# AI-Driven Image Analysis for Nanofiber Characterization: From Diameter Measurement to Multiparameter Assessment

**DOI:** 10.1021/acsomega.5c12433

**Published:** 2026-05-16

**Authors:** Serdar Tort, Haticenur Negiz, Emre Tunçel, Güliz Demirezen, Mustafa Umut Demirezen

**Affiliations:** † Department of Pharmaceutical Technology, Faculty of Pharmacy, 37511 Gazi University, Ankara 06330, Türkiye; ‡ Turkish Medicines and Medical Devices Agency, Ankara 06520, Türkiye; § Artificial Intelligence Policies Association (AIPA), Ankara 06800, Türkiye; ∥ 420479 Huawei R&D Center, MSDC Department, İstanbul 34800, Türkiye

## Abstract

The high surface area and porosity of the nanofibers enable a wide range of pharmaceutical applications, including tissue scaffolds, wound dressings, and drug-loaded films, as well as applications in other fields such as energy, electronics, and environmental remediation. The properties of nanofibers are directly dependent on the production parameters, such as applied voltage, solution flow rate, polymer concentration, solvent type, and collector distance, and there is a complex interplay between these parameters that makes their optimization challenging. Therefore, accurate determination of nanofiber properties, especially fiber diameter, is essential for quality control, process optimization, and functional performance assessment. This review systematically investigates computational methodologies employed in the characterization of nanofibers, with a particular focus on the measurement of fiber diameter. Initially, manual measurements and open-source tools such as DiameterJ, GIFT, and SIMpoly are described, highlighting their advantages and limitations. Subsequently, artificial intelligence-based strategies are described, ranging from classical machine learning models to deep learning architectures, as well as more advanced approaches such as generative frameworks and transformer-based models. In addition, comparisons with traditional characterization methods, industry applications including smart manufacturing, and automated quality control are outlined. Finally, the review examines emerging and prospective artificial intelligence methodologies in the analysis of nanofibers, offering conclusions and recommendations.

## Graphical abstract

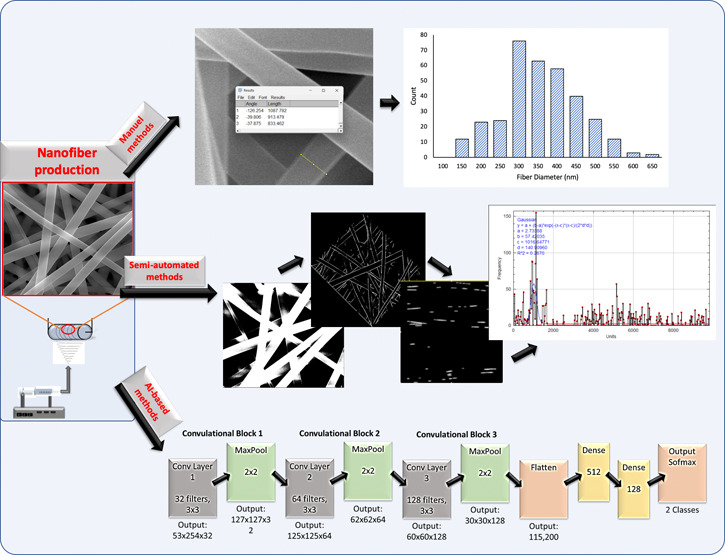

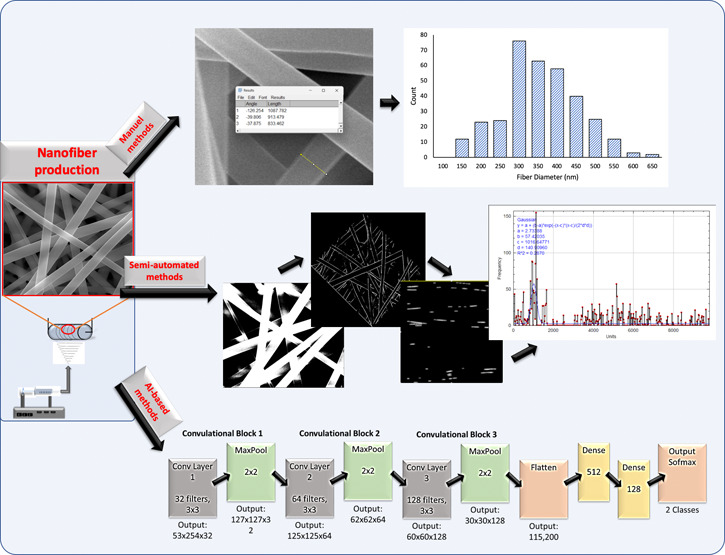

## 1 Introduction

Electrospinning is an efficient technique for producing micro- and nanofibers from synthetic or natural polymer solutions. Nanofibers have revolutionized applications in filtration, energy storage, and biomedical engineering due to their high surface area and tunable porous structures.^1^ Recent studies on electrospinning have led to the development of nanoparticles composed of hybrid nanofibers, as well as new techniques such as coaxial and triaxial electrospinning, enabling the production of core–shell or multilayered fibers with enhanced properties.^2–4^ In the electrospinning process, an electric field is applied to a solution or melt of polymers, leading to the formation of a charged jet that stretches and thins due to electrostatic repulsion and solvent evaporation. After that, nanofibers are collected on a grounded collector with unique morphological and functional characteristics.

The main parameters that affect nanofiber characteristics include the properties of the polymer solution (concentration, viscosity, and conductivity), process parameters (applied voltage, flow rate, and needle tip-to-collector distance), and environmental factors (humidity and temperature). The diameter, morphology, and surface characteristics of the fibers can be adjusted to be personalized for specific applications. The diameter of the nanofiber is particularly sensitive to these parameters. For example, increasing the concentration or viscosity of polymers often results in thicker fibers due to enhanced chain entanglements, whereas low-viscosity solutions can lead to bead formation rather than uniform fibers. The applied voltage also plays an important role, as higher stress can prolong the force on the polymer jet, leading to thinner fibers. However, very high voltages can cause jet instabilities and bead formation. The flow rate of the polymer solution also affects fiber diameter: higher rates often produce thicker fibers due to increased material, while lower rates produce smoother fibers with improved uniformity. The needle tip-to-collector distance affects fiber formation by impacting the solvent evaporation rates. Shorter distances may result in insufficient solvent evaporation and nanofiber solubilization, whereas optimal distances enable uniform nanofiber deposition. Additionally, environmental factors, such as humidity and temperature, can alter the solvent evaporation rate, fiber solidification, and overall morphology.

Accurate characterization of nanofiber mats—including fiber diameter, pore size, porosity, and alignment—is crucial for optimizing performance.^5^ Traditionally, researchers relied on manual or semiautomated image analysis (e.g., measuring diameters on scanning electron microscopy images by hand or using basic thresholding). However, these approaches are time-consuming, subjective, and often inconsistent, especially for dense or overlapping fibers.^6,7^ For instance, manually analyzing a single scanning electron microscopy (SEM) image can take hours, and results can vary by the operator. Semiautomated tools such as DiameterJ, an ImageJ plugin, represented a significant advance by analyzing an image in approximately 20 s and achieving diameter measurements within 2% of known values much faster than manual methods. Moreover, DiameterJ can sample orders of magnitude more fibers than a human operator, thereby greatly improving the statistical reliability.^8^

Despite such progress, early automated methods still faced limitations. Standard image processing struggles when fibers are highly entangled or the contrast is low.^7^ Parameter tuning (e.g., for edge detectors or threshold levels) can be data set-specific, reducing generality. Moreover, traditional porosimetry techniques (e.g., BET surface area analysis) require destructive testing and lengthy procedures.^5^ By contrast, image-based porosity estimation offers a rapid, nondestructive alternative,^5^ though its accuracy historically lagged physical methods.

These drawbacks have motivated the integration of artificial intelligence (AI) in nanofiber characterization to enhance the accuracy, speed, and automation. Techniques such as machine learning (ML) and deep learning (DL) offer rapid, automated, and more robust alternatives for assessing fiber diameter and other morphological parameters, even under complex imaging conditions. Beyond fiber diameter, comprehensive nanofiber characterization encompasses several interconnected morphological parameters that collectively determine material performance. Porosity, defined as the ratio of void volume to total volume, directly influences filtration efficiency, cell infiltration in tissue scaffolds, and drug release kinetics.^9^ Pore size distribution affects molecular transport and cellular behavior, with smaller pores providing higher filtration efficiency but potentially limiting nutrient diffusion in biomedical applications.^5^ Fiber alignment and orientation significantly affect mechanical properties, with aligned fibers exhibiting higher tensile strength in the fiber direction than randomly oriented mats.^10^ Recent advances in AI-based image analysis have enabled simultaneous quantification of these parameters from a single SEM image, providing a more holistic understanding of nanofiber mat characteristics.^7^

This review addresses not only diameter measurement but also extends the discussion to AI-driven approaches for porosity estimation, pore size analysis, and fiber orientation quantification, thereby providing researchers with a comprehensive toolkit for nanofiber characterization. In addition to characterization, AI is increasingly contributing to process optimization, facilitating predictive modeling, and real-time control of electrospinning parameters to achieve consistent fiber diameters tailored to specific applications. This review aims to summarize traditional and AI-driven computational methodologies for nanofiber characterization, highlight their advantages and limitations, and discuss future directions for integrating advanced approaches into research and industrial applications.

## 2 Conventional (Non-AI) Approaches for Nanofiber Diameter Measurements and Other Characterizations

The diameter of the nanofibers is typically determined from images obtained by optical microscopy, scanning electron microscopy, transmission electron microscopy (TEM), or atomic force microscopy (AFM).^11^ Diameter measurements can be performed by processing the collected images using manual or automated methods. The methods used for nanofiber diameter measurement are described in detail below, and different conventional approaches for nanofiber diameter analysis are compared in Table 1.

**Table 1:** Comparison of Conventional Nanofiber Diameter Measurement Methods

Method	Measurement Principle	Advantages	Limitations	Example applications
**Manuel Measurement**	Fiber diameter is manually measured from images using a known scale	• Simple	• Operator dependency	^37–40^
		• Widely accepted method	• Low throughput	
		• No complex preprocessing required	• Limited number of measurements	
			• Time-consuming	
**Distance Transform Method**	Diameter calculated as twice the distance between skeleton pixels and nearest fiber edge in a binary image	• Widely used automated method	• Sensitive to thresholding and skeletonization	^41–43^
		• Enables large number of measurements (>1000), suitable for statistical analysis	• Intersection points may cause errors	
**DiameterJ**	It is based on the distance transform and performs superpixel-based diameter calculation through segmentation and skeletonization	• Rapid, semiautomated method, validated, open-source tool	• Sensitive to image resolution and magnification	^44–62^
		• Reduces operator bias	• Requires fibers ≥10 px in diameter	
		• Provides fiber diameter distribution, mesh hole analysis, and fiber orientation evaluation	• Segmentation errors, fiber overlap, and multimodal diameter distributions may reduce accuracy^8^	
**Direct Tracking Method**	The method determines fiber diameter by pixel counting perpendicular to fiber orientation after binary thresholding and horizontal–vertical image scanning	• Enables automated	• Requires accurate binarization	^25,26^
		• Enables large number of measurements (>1000), suitable for statistical analysis	• Intersection points may cause errors	
		• Provides fiber orientation information		
**Radon Transform Method**	Detects fiber edges using Canny edge detection and estimates diameter via normalized Radon transform projections	• Allows diameter measurement without isolating individual fibers	• Parameter tuning required for different resolutions	^63^
		• Can be adapted to different image resolutions through parameter adjustment		
**SIMPoly**	Uses edge detection, skeletonization, and centerline-to-edge distance calculation for semiautomated diameter estimation	• Provides statistical measures, histograms, color maps	• Semiautomated	^64–67^
		• Provides faster analysis, higher accuracy, and less human bias compared to manual measurements and DiameterJ	• Requires MATLAB	
			• Limited accessibility compared to ImageJ-based tools	
**GIFT**	Uses edge detection and morphological filtering for segmentation and estimates fiber diameter by statistically evaluating distances between parallel or repeating fiber structures	• Provides high accuracy and consistency	• Performance may be reduced in blurred SEM images, highly textured fiber surfaces, or images with strongly different fiber diameters	^68–73^
		• Performs well in complex images with overlapping fibers or varying contrast	• Hidden fiber edges cannot be measured	
		• Reduces user bias through statistical evaluation		

### 2.1 Manual Diameter Measurement

The most common method used for diameter detection is manual measurement. For the manual measurement, nanofiber diameters can be determined proportionally to a known length scale using open-source software such as ImageJ and FIJI (Figure 1).^12^ The measurement process is carried out by manually drawing the diameter of the nanofiber in the image. As a result, it has drawbacks such as interoperator variability, significant measurement error, and a limited number of measurements.^13^

**Figure 1:**
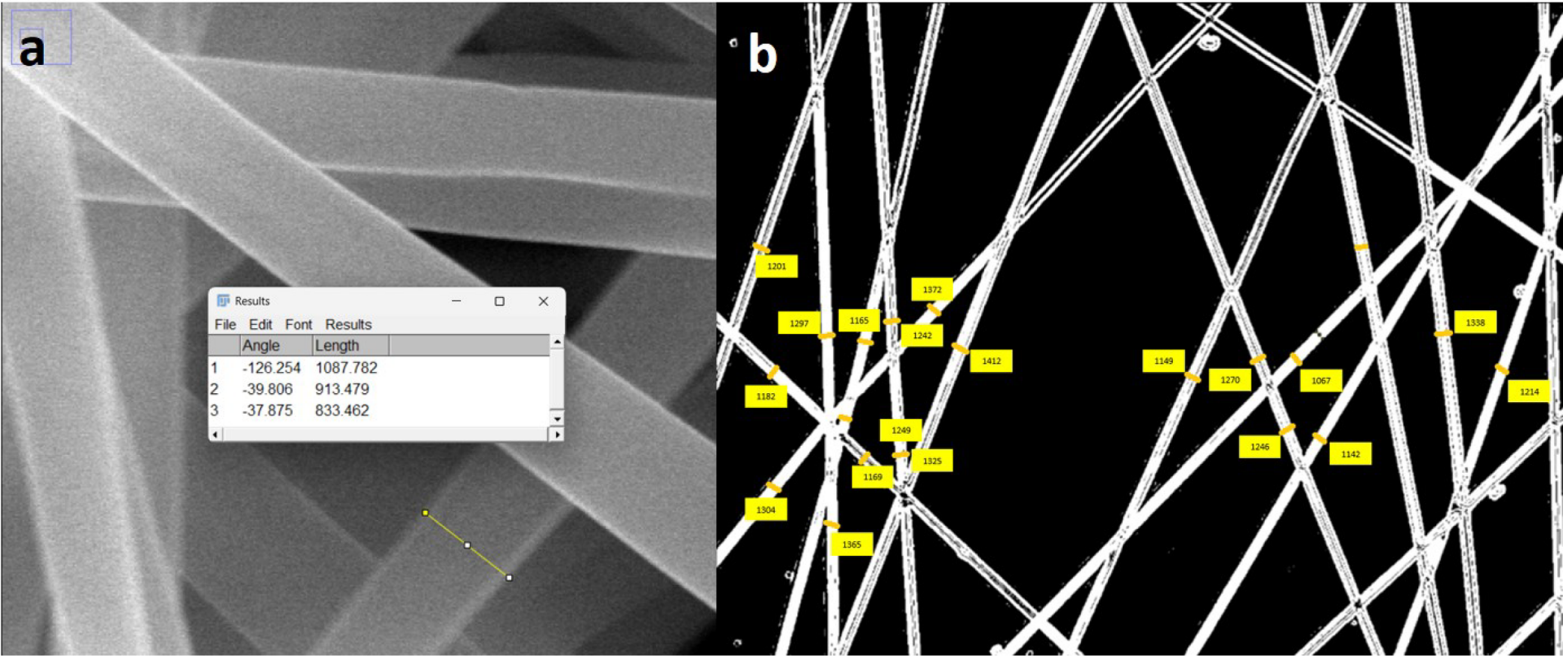
Manual fiber diameter measurement workflow using ImageJ software. Measurement lines (yellow) drawn by the operator and measurement values obtained relative to the reference scale (a) on the SEM image and (b) on the optical microscope image of the electrospun nanofiber mat.

### 2.2 Distance Transform Method

Although various methods have been defined in the literature, the most used automated method is the distance transform method. The distance transform method is a methodology for calculating the distance between each pixel and the nearest nonzero pixel in an image. First, to create a binary image, pixels above a certain threshold value are converted to 1, and those below are converted to 0. This process clarifies the object’s and background boundaries. The center point of the object is determined by the skeletonization process. To determine the center point of the object, points that are equidistant from the object’s boundary are taken. Thus, an object skeleton with a single-pixel thickness is created. Diameter measurement is performed by determining the distance from a pixel on the skeleton to the nearest nonzero pixel.^14,15^

In the literature, studies have been carried out on increasing the accuracy of the measurements by making various modifications to the distance transform method. The measurement error was reduced by removing the intersection points during skeletonization.^15–18^ The Canny edge detection technique was used to determine nanofiber edges with higher accuracy during thresholding.^16^ In most cases, the distance transform method allows for more than 1000 measurements per image.

Ziabari et al.^19^ examined the diameter measurement and production process of nanofibers produced by electrospinning. In the study, the effects of four main parameters, which are concentration, distance, applied voltage, and volumetric flow rate, on fiber properties were investigated using Response Surface Methodology (RSM). For this purpose, poly­(vinyl alcohol) was dissolved in distilled water, and the fiber was spun by varying aforementioned parameters. The researchers determined fiber diameter from SEM images using the New Distance Transform method, which demonstrated greater efficacy than the direct tracking and distance transform methods they developed. The results of the study showed that the selected four parameters significantly affect fiber diameter and uniformity, and by controlling these parameters, fiber diameters can be optimized.

#### 2.2.1 DiameterJ

DiameterJ is a semiautomated open-source plugin that utilizes the distance transform method and runs on ImageJ/FIJI. DiameterJ generates histograms using the distance transform approach, while for studies that require a single value, it utilizes a value termed “Superpixel,” which is derived by dividing the total fiber area in the image by the total center lengths. It is employed for mesh hole analysis and fiber orientation identification, in addition to automatically performing the segmentation required for diameter measurement (Figure 2).^8^

**Figure 2:**
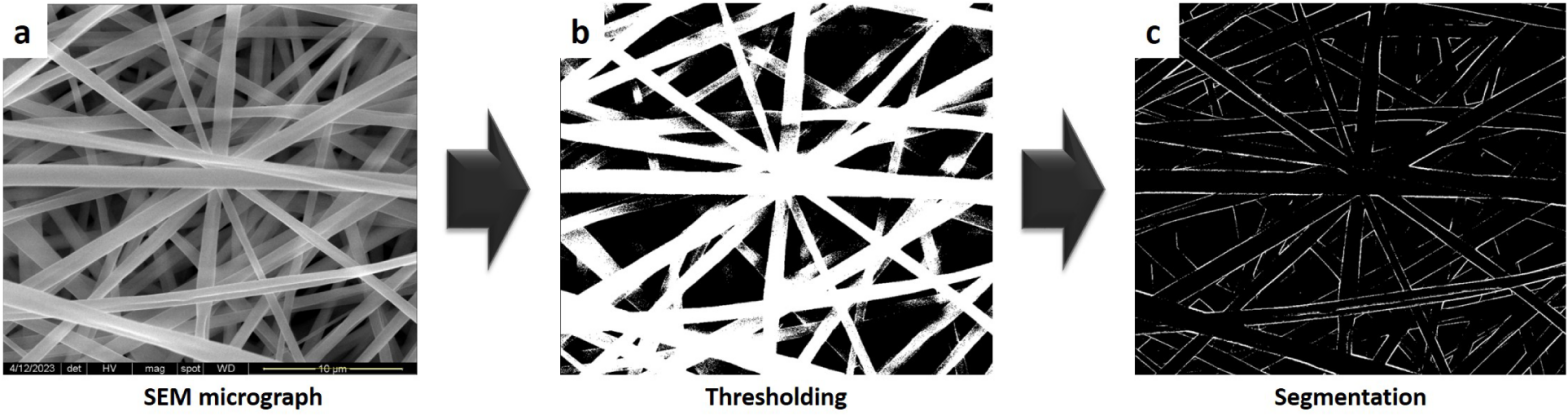
DiameterJ semiautomated image segmentation processing workflow for nanofiber diameter analysis: (a) original SEM micrograph input, (b) binary segmentation result after thresholding, where white pixels represent fiber regions and black pixels represent the background, and (c) skeletonization output showing fiber centerlines (single-pixel width).

García-Hernandez et al. produced hydrolyzed collagen and poly­(vinyl alcohol) (PVA) nanofiber membranes using electrospinning at two different distances.^20^ The SEM images were analyzed for the fiber diameter measurement using the DiameterJ plugin. The results showed that the fiber diameter distribution ranged from 360 to 960 nm depending on the electrospinning distance. Friuli et al. developed controlled-release tablet formulations containing meloxicam and carvedilol using polymeric nanofibers and analyzed SEM images of nanofibers using DiameterJ.^21^ Another study using nanofibers as a drug delivery system investigated the production of PVA-carboxymethyl cellulose (CMC) nanofiber mats containing zinc oxide nanoparticles and erythromycin for antibacterial and wound dressing applications.^22^ SEM analyses of the fibers were performed for characterization of their morphological properties, and fiber diameter measurements were found to be approximately 200 nm by DiameterJ. In a study conducted by Sadeghi et al., nanofiber scaffolds containing polycaprolactone (PCL), chitosan, and polypyrrole (PPy) were produced by electrospinning.^23^ Different properties of the nanofibers obtained including diameter measurement, fiber length, average pore area, porosity percentage, and number of intersections, were analyzed with DiameterJ. The average diameter of all fibers was found to be in the range of 30–180 nm. In another study on tissue engineering, researchers investigated the creation of cell sheets with the requisite arrangement using electrospinning in tissues that necessitate aligned cells, such as muscle, nerve tissue, and cartilage.^24^ For this purpose, fibers were obtained by using PCL and heat-sensitive poly­(*N*-isopropylacrylamide) (PNIPAAm) in different ratios. The orientation and diameter of the fibers were determined using OrientationJ and DiameterJ, respectively. The findings indicated that the diameter of the fibers ranged between 1 and 3 μm, and the orientation index of all fibers exceeded 0.65.

### 2.3 Direct Tracking Method

The direct tracking method can be performed on a binary image, like the distance transform method. After the thresholding process, the direct tracking method algorithm scans the image in horizontal and vertical directions. For the horizontal scan, the algorithm searches for the first white pixel (nanofiber) adjacent to a black pixel (background). It then counts the number of white pixels until it finds the next black pixel. This shows the fiber’s width in the horizontal direction. For the vertical scan, starting from the center of the horizontal scan, the algorithm counts the number of white pixels in the vertical direction until it encounters a black pixel. This determines the width of the fiber in the vertical direction. Based on these two results, the nanofibers’ orientation is determined. The diameter is calculated using the number of pixels counted in the direction perpendicular to the nanofiber’s orientation. Similar to the distance transform method, fiber identification can be used to extract the intersection areas of nanofibers and improve measurement accuracy.^25^ It was reported that more than 1000 measurements can be performed using the direct tracking method.^26^

### 2.4 Radon Transform Method

In this method, defined by Öznergiz et al., the edges are identified by first applying the Canny edge detection process. Subsequently, the normalized Radon transform is used to obtain projections of the image at different angles.^27^ The Radon transform is a mathematical method that determines the integral of a function along rays. It is widely used in tomographic systems, such as X-ray imaging in medical applications, to reconstruct two- or three-dimensional objects from acquired integrals.^28^ It was reported that this method works without isolating each fiber, which is a common source of mistakes in other methods, and the Radon transform method can be further improved by adjusting parameters for different image resolutions.^27^

### 2.5 SIMPoly

Murphy et al. developed SIMPoly (Semiautomated Image Measurements of Polymers), a MATLAB-based semiautomated method for quantifying nanofiber diameters in SEM images. The algorithm improves image contrast, reconstructs fiber morphology, and isolates fibers via edge detection. After that, similar to the distance transform method, the algorithm skeletonizes the fibers to extract centerlines and calculates diameters by using the shortest distance between each centerline pixel and the fiber edge. The output consists of statistical measures, histograms, and color maps for visualization. It was reported that SIMPoly provides faster analysis, higher accuracy, and less human bias than manual measurements and semiautomated DiameterJ approaches, making it a reliable tool for nanofiber characterization.^29^

### 2.6 GIFT

GIFT (General Image Fiber Tool), developed by Götz et al., is a freely available open-source macro that runs on ImageJ/FIJI that automates the image analysis approach for evaluating nanofiber diameters in SEM images. The algorithm of GIFT uses edge detection and morphological filtering for segmentation. In contrast to distance transform, the algorithm basically measures the distance between parallel lines in the image, and then, it statistically evaluates to calculate fiber diameters. Unlike approaches that rely on grayscale values, GIFT isolates and counts distances between repeating structures, ensuring accurate recognition even in complicated images with varying contrast, overlapping fibers, or grayscale gradients (Figure 3). In the comparative analysis by Götz et al., it was reported that GIFT outperformed DiameterJ and manual measurements in terms of accuracy and consistency, especially under demanding imaging settings. It was claimed that its statistical method reduces user bias while increasing reliability, making it an effective tool for nanofiber characterization.^12,30^

**Figure 3:**
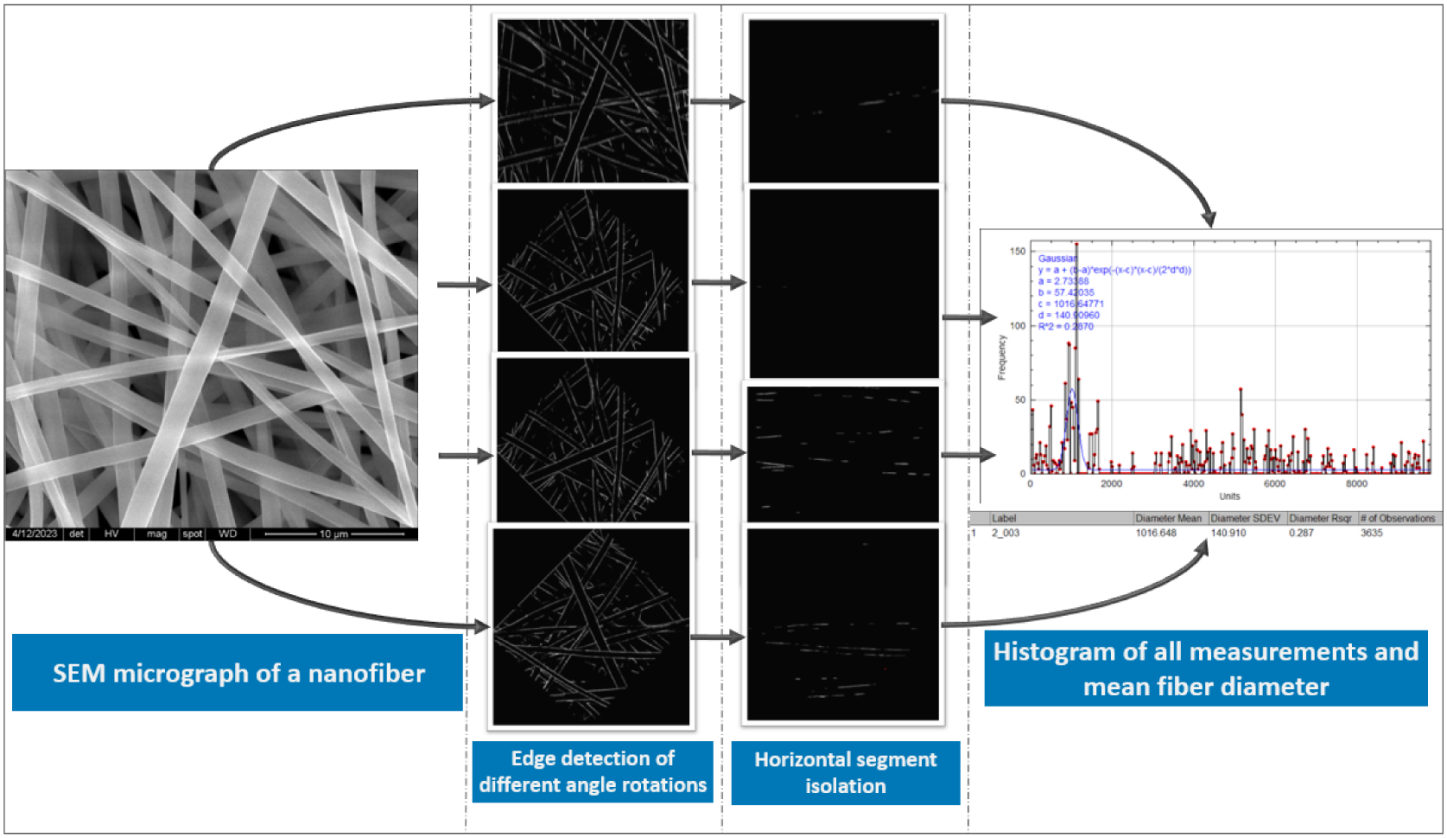
General Image Fiber Tool (GIFT) analysis pipeline for automated fiber diameter quantification. The algorithm employs edge detection and morphological filtering for segmentation, followed by statistical analysis of parallel line distances.

In another study by Götz et al., the aim was to detect fiber-level defects in nanofiber nonwoven materials under tensile stress by recording acoustic emission (AE) signals using a highly sensitive sensor.^31^ For fiber diameter measurements, the GIFT software was used, enabling unbiased analysis without any preselection of fibers in the images. In the study, invisible defects were detected by correlating stress–strain curves and AE signals of poly-l-lactide acid nanofibers produced in different diameters: small, medium, and large. The results demonstrated the effectiveness of the method and its potential for quality assurance of nanofiber materials, including medical applications.

Another study using the GIFT method for fiber diameter measurement was performed by Götz et al. (2024).^32^ The study described two distinct patented bicuspid valve designs for chronic venous insufficiency and evaluated their performance. The designs are made of electrospun thermoplastic silicone polycarbonate polyurethane nanofiber leaflets attached to a nitinol stent. Fiber diameter measurements were performed utilizing the GIFT method on SEM images, thereby assessing the quality of the produced nanofibers. The valves were examined under physiological pressure, and the results of the study showed that both valves largely met the accepted requirements.

### 2.7 Other Diameter Measurement Methods

Various open-source and commercial software were developed for the automated measurement of nanofiber diameters, such as SEMAnalyzer, FibreQuant,^13^ Nano Measure,^33^ FiBar,^34^ Fiber Pro, Fiber Thickness App, and Phenom FiberMetric.^30^ Although there is not much information available regarding specific algorithms employed in this software, automated fiber diameter measurement tools typically use image processing methods to examine micrographs from SEM analysis. By considering the limitations of manual measurements, these techniques aim to deliver quick and accurate results. A comparison of different conventional methods for measuring nanofiber diameters is presented in Table 1.

In addition to measuring fiber diameter, various methods were proposed for the segmentation and edge detection processes required for the measurement. For the segmentation, global thresholding,^29^ machine learning,^35^ or edge detection techniques can be employed. Although the Canny edge detection method is preferred for edge detection, the Sobel filter was also suggested.^13^ In addition, it was reported that there are various algorithms that can be used for the edge detection process.^36^

### 2.8 Other Characterization Methods

Other important parameters used in the optical characterization of nanofibers are orientation and porosity. Various approaches and methods have been developed for orientation analysis. As in fiber diameter analysis, manual measurements can be performed relative to a reference axis using image analysis software such as ImageJ. One of the best-known conventional semiautomated methods is the Hough Transform method, which is based on the principle of determining a straight line and its alignment by tracking pixels that fit a mathematical equation defining a line with a constant angle.^74^ Besides using various algorithms, such as Fast Fourier Transform (FFT) and Direct Tracking, alignment analysis can also be performed using open-source macros like OrientationJ.^8,75^

Porosity of nanofibers in SEM micrographs can be measured using ImageJ and its various plugins. The main principle of porosity measurement is to generate binary images using global or adaptive threshold algorithms (such as Otsu’s method) and then calculate the ratio of black pixels (void area) to the total number of pixels (void + solid area). The image analysis methods only provide 2D porosity measurements.^9^ Physical methods have also been proposed for 3D porosity measurements.^76^

## 3 AI-Driven Approaches for Diameter Measurement and Other Characterizations

AI-driven approaches, particularly those based on machine learning and deep learning, can learn complex features of nanofiber images that are difficult to hand-code.^6,7^ In the era of Industry 4.0 and smart manufacturing, such methods enable real-time monitoring and quality control of nanofiber production.^77^ AI-based methods have demonstrated clear advantages over traditional manual and rule-based techniques in nanofiber characterization. A primary benefit is improved accuracy and objectivity.^8^ Compared with conventional image-analysis workflows, AI-assisted methods can improve reproducibility, throughput, and multiimage processing in nanofiber analysis.^7,8,78^ However, interpretability is not solved automatically and often requires an explicit explainable AI tool.^79–82^ Over roughly the past five years, there has been a surge in AI applications for analyzing electrospun nanofibers and other fibrous materials. These include machine learning models for defect detection,^77^ convolutional neural networks for fiber segmentation, and even generative models to create synthetic fiber images for training data augmentation.^7^ Studies demonstrate that automated and AI-assisted techniques can markedly improve the throughput and reproducibility of nanofiber measurements,^8,78^ and deep-learning-based diameter computation can achieve low error on complex fiber micrographs.^7^ An overview of the AI-assisted nanofiber characterization workflow is represented in Figure 4.

**Figure 4:**
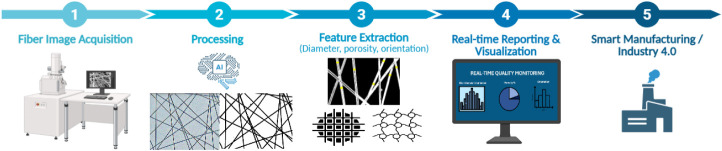
Overview of AI-assisted nanofiber characterization workflow. The process begins with SEM image acquisition, followed by preprocessing (noise reduction, contrast enhancement). AI models ranging from classical machine learning for parameter prediction to deep learning for semantic and instance segmentation analyze the images to extract morphological parameters. Based on the output metrics, this automated pipeline enables high-throughput quality control and real-time process monitoring in smart manufacturing environments (this figure was created with biorender.com).

### 3.1 Classical Machine Learning (ML)

Prior to the deep-learning era, researchers applied classical ML algorithms to analyze features extracted from nanofiber images. Common approaches included training classifiers such as support vector machines (SVMs), random forests (RFs), k-nearest neighbors (k-NN), or decision trees on descriptors such as fiber diameter distribution, fiber orientation, and texture features. For example, one study used an SVM and linear discriminant analysis (LDA) in conjunction with an artificial neural network to classify electrospun fiber images as defect-free or defective.^77^

Classical ML models such as SVMs, k-NNs, and decision trees have been used to predict nanofiber diameters, often by learning the relationship between electrospinning parameters and the resulting fiber size. Nurwaha and Wang measured fiber diameters from SEM images (using ImageJ) and trained an SVM-based model to predict the mean fiber diameter; the SVM outperformed an adaptive neuro-fuzzy model and gene-expression programming in accuracy.^83^ In such studies, ML predictions show strong agreement with experimental diameters, achieving high coefficients of determination (*R* ^2^ = 0.92–0.94).^84,85^ These approaches can significantly speed up diameter estimation compared to manual image measurements, which are labor-intensive and time-consuming.^86^ Traditional image processing tools (e.g., thresholding algorithms or ImageJ plugins like DiameterJ) can automate diameter measurement; however, their reliability varies with image quality.^87^ By contrast, studies have found that ML regression models (e.g., decision trees or random forests) can match or exceed the consistency of manual and basic image-processing methods for diameter analysis.^83,85^ In short, classical ML models provide a viable alternative to manual tracing by quickly predicting fiber diameters with comparable accuracy.

Classical ML techniques have been applied to identify anomalies or defects (such as bead formation or nonuniform regions) in nanofibers. For example, Ieracitano et al. (2021) use a hybrid scheme: first, an unsupervised autoencoder extracts feature representations of SEM image patches, and then a shallow neural network classifies each patch as defect-free or defective. This method successfully detected structural anomalies in electrospun fibers, achieving around 92% classification accuracy. It also outperformed conventional standalone algorithms (including standard supervised classifiers) and even some deep convolutional neural network (CNN)-based strategies while using a smaller, more interpretable model.^77^

Classical ML techniques have also been applied to characterize structural properties of nanofibers, such as porosity and pore size distribution. Typically, porosity is first quantified by digital image analysis (e.g., binarizing SEM images to identify void areas) and then related to processing conditions via ML. Cuahuizo-Huitzil et al. demonstrated that image-derived porosity (2D digital porosity) of electrospun veils correlates well with conventional measurements, validating image processing as a quick estimation method.^9^ Building on such data, regression models (like random forests or multiple linear models) have been trained to predict porosity from fabrication parameters. For instance, one study compiled a data set of polymer concentrations, voltages, and other inputs and used a random forest to predict not only fiber diameter but also whether the fiber mat would have acceptable uniformity. The random forest achieved accurate predictions across both tasks, highlighting its ability to capture complex parameter–structure relationships.^88^

Classical ML has been explored to predict mechanical properties of nanofiber assemblies (e.g., tensile strength, Young’s modulus) based on image-derived morphological features. The rationale is that fiber morphology—diameter, alignment orientation, fiber packing density, etc—strongly influences mechanical performance.^10,86^ By quantifying these features from microscopy images, one can train regression models to forecast how a nanofiber mat will behave mechanically. For example, studies on fiber-reinforced composites have used features such as fiber orientation distribution (from SEM) to predict composite stiffness using regression trees or SVMs, demonstrating that ML can map microstructure to macro properties.^89^ In electrospun nanofibers, smaller fiber diameters and aligned fiber arrangements generally increase tensile strength and modulus by reducing stress concentrators and enhancing load transfer.^10^ ML models can capture such correlations; for instance, they may learn that a decrease in average fiber diameter (along with low diameter variation) and a high alignment index leads to higher predicted strength. More broadly, machine-learning studies in composite and materials-imaging literature suggest that microscopy-derived orientation and morphology features can be linked to mechanical properties, although direct evidence for electrospun nanofiber mats remains limited.^89,90^

### 3.2 Deep Learning (DL)

In contrast to classical approaches, CNNs and related deep models have become the dominant techniques for image analysis,^91^ making them adept at handling complex fiber networks. The basic architecture of CNNs for nanofiber image classification is illustrated in Figure 5.

**Figure 5:**
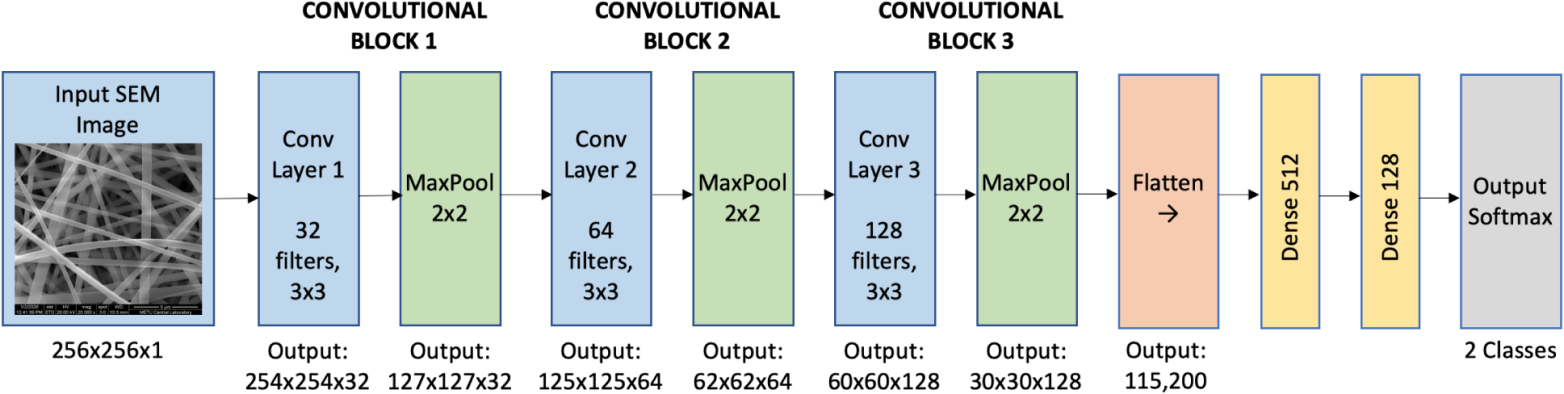
Basic architecture of a convolutional neural network (CNN) for nanofiber image classification. The input SEM image is processed through alternating convolutional and pooling layers that progressively extract hierarchical features from low-level edges and textures in early layers to high-level structural patterns in deeper layers. Fully connected layers at the end perform classification based on the learned feature representations.

CNN-based architectures, particularly U-Net, have been widely adopted to identify fiber pixels in images using semantic segmentation. Originally developed for biomedical microscopy, U-Net excels at delineating structures with limited data by using an encoder-decoder with skip connections. In nanofiber applications, U-Net variants have achieved high-fidelity fiber segmentation even when fibers overlap, enabling precise diameter measurements across entire images (Figure 6).^7^ For instance, Huarachi et al. trained a modified U-Net to regress a distance map for each fiber, allowing separation of touching fibers and accurate diameter computation; their model obtained low mean absolute error on fiber diameters, demonstrating only minor deviation from ground truth measurements.^7^ Fully convolutional networks (FCNs) like U-Net operate on the whole image and classify each pixel as fiber or background (or even individual fiber instance), producing segmentation masks from which diameters and pore sizes can be quantified. U-Net and other FCN architectures remain popular for nanofiber segmentation tasks due to their pixel-level precision. Recent studies have explored enhancements like residual connections and multiscale feature fusion to handle fibers of varying thickness. Yu et al. introduced a revised U-Net model for nanofiber sizing that processes multiple images in batch—a notable improvement since earlier MATLAB—based tools could not batch-process images.^78^ By training on many images at once, they improved the consistency of diameter distributions measured across an entire sample set. Deep architectures are also extended to 3D data (e.g., fibrous structures in tomography) with volumetric CNNs, although 2D SEM analysis remains the primary focus.

**Figure 6:**
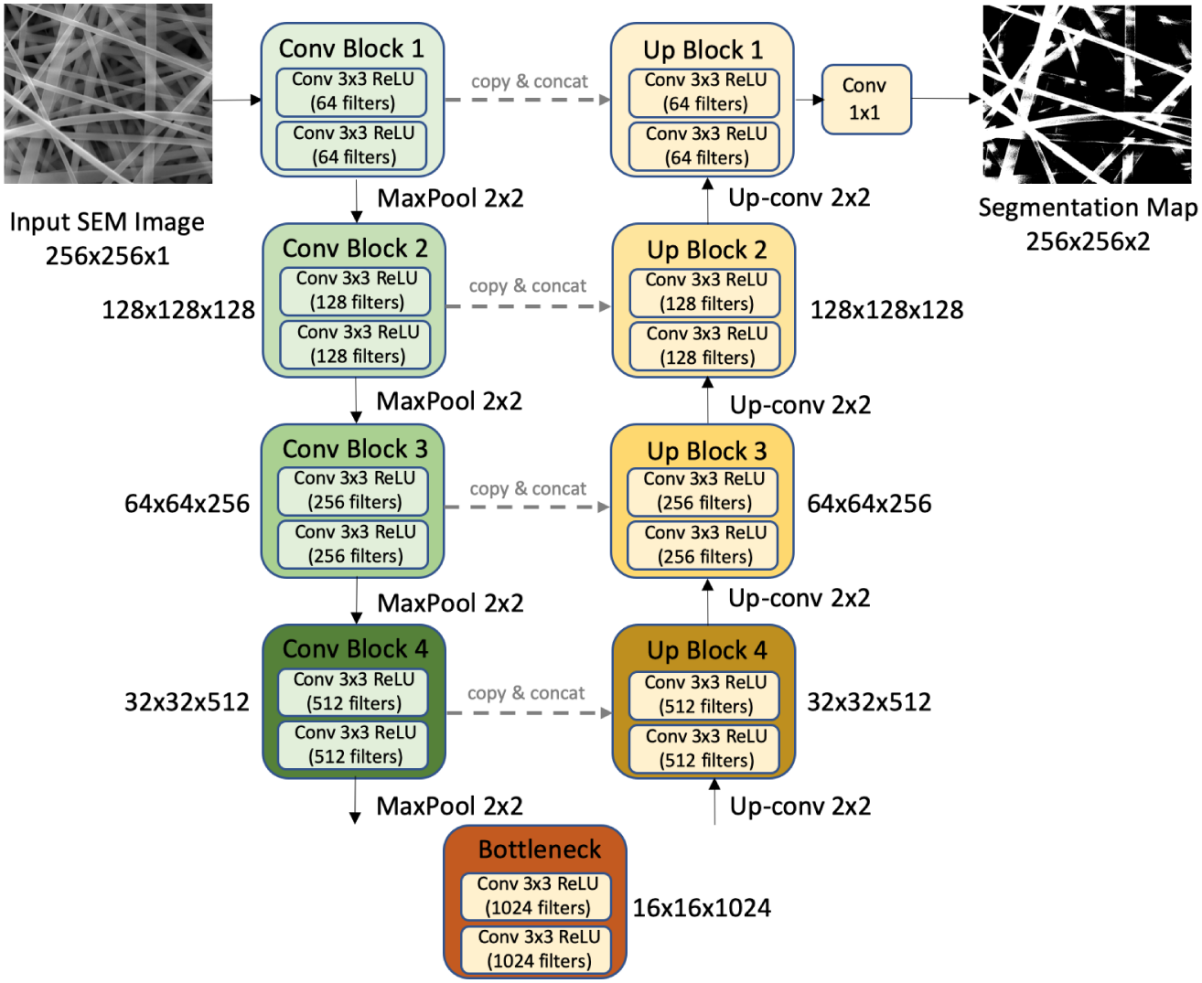
U-Net architecture for semantic segmentation of nanofiber images. The encoder path (left, descending) progressively reduces spatial resolution while increasing feature depth through convolutional and max pooling operations. The decoder path (right, ascending) uses transposed convolutions to restore spatial resolution. Skip connections (horizontal arrows) concatenate encoder features with decoder features at corresponding resolutions, preserving fine spatial details essential for accurate fiber boundary delineation. The final 1 × 1 convolution produces a pixel-wise segmentation map classifying each pixel as fiber or background.

Beyond pixel-level classification, instance segmentation methods have been explored to distinguish individual fibers rather than treating all of the fibers as a single class. Mask R-CNN, for example, was utilized to detect and segment short fibers in composite materials.^6^ Rahman (2021) reported that a Mask R-CNN-based system could simultaneously identify, segment, and measure fibers in SEM images, even under significant overlap, after training on simulated fiber images. Compared with classical approaches such as the Hough transform or morphological algorithms, the CNN-based methods reduced the error in length and orientation measurements for each fiber and were robust to noise and fiber curvature.^6^

DL is also increasingly used to identify structural defects in nanofiber mats, including beads, breaks, and contamination.^77^ In general, deep CNNs offer greater accuracy and automation than classical ML, at the expense of requiring larger annotated data sets and higher computational resources.

#### 3.2.1 Comparisons with Classical ML

Studies that directly compare classical ML and DL find that each has pros and cons (Figure 7). Classical methods often require manual feature extraction but can excel with limited data and offer faster training and inference. Deep learning can automatically extract complex features (e.g., recognizing subtle texture differences in fiber mats) and often achieves higher accuracy given enough data; however, it is computationally intensive and can be a black box.^92,93^ Classical ML models such as SVMs or random forests are generally quicker to train and tune, and they generalize well on small or moderate-sized data sets, making them attractive for nanofiber studies, where data are often limited. In terms of accuracy, deep learning models slightly edge out classical ones in some reports (e.g., CNN-based classifiers might catch faint defects that an SVM misses), but the difference can be marginal when feature engineering for the classical model is strong.^77^

**Figure 7:**
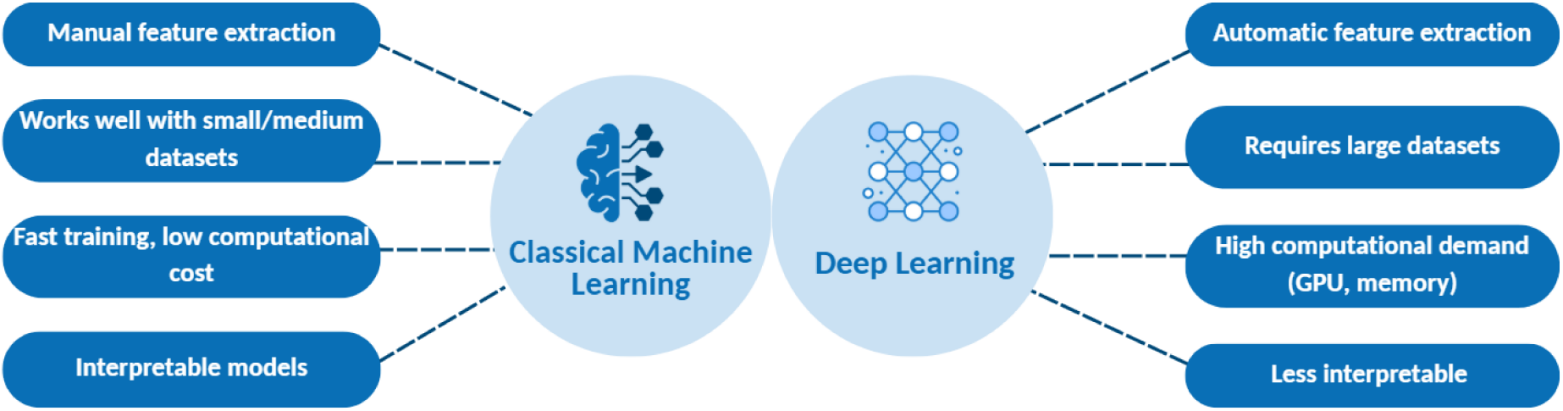
Comparative overview of classical machine learning (ML) vs deep learning (DL) approaches for nanofiber image analysis. Classical ML methods (left) require manual feature extraction (e.g., texture descriptors, histogram statistics) before training models such as SVM or Random Forest for classification or regression tasks. DL approaches (right) use convolutional neural networks that automatically learn hierarchical feature representations directly from raw images. While classical ML offers faster training and better interpretability with limited data, deep learning achieves higher accuracy for complex segmentation tasks when sufficient training data are available (this figure was created with biorender.com).

For computational efficiency, classical methods are usually less demanding: they do not require graphics processing units (GPUs), and inference can be performed in real time on standard computers, which is beneficial for inline quality control. Deep models require more memory and computation, though once trained, a lightweight CNN can also run quickly if optimized. For generalization, classical models are often more interpretable across changing data distributions, while deep models may need retraining but can capture more invariances.

Overall, well-tuned classical algorithms remain competitive for structured tasks such as fiber diameter measurement or basic defect classification; in contrast, deep learning offers higher performance ceilings for complex image analyses. Hybrid approaches that use CNNs for feature extraction or segmentation combined with classical classifiers are also promising as they integrate the strengths of both paradigms.

#### 3.2.2 Learning Strategies—Transfer Learning and Self-Supervised Learning

In nanofiber analysis, data are often limited (since obtaining and labeling high-resolution SEM/TEM images is labor-intensive). To address this, researchers employ transfer learning and self-supervised learning to boost model performance.

Transfer learning typically means starting with a CNN that was pretrained on a large data set (like ImageNet) and fine-tuning it on nanofiber images. This leverages learned low-level features (edges, textures) that are somewhat universal, thus requiring fewer fiber-specific images to achieve good results. Guan and Zou used transfer learning with pretrained GoogleNet and ResNet CNNs to classify electrospun fiber images by morphology. The pretrained models, when fine-tuned on fiber polarization images, significantly improved classification accuracy over training from scratch—reaching up to ∼96% accuracy, versus ∼79% using conventional single-image features.^94^

Self-supervised learning is another promising approach. Obtaining labeled training data (e.g., pixel-wise fiber annotations) is a major bottleneck. Self-supervised learning offers a solution by leveraging unlabeled data to pretrain models. For nanofibers, one could employ techniques such as image inpainting (removing a fiber segment and tasking the model to reconstruct it) or contrastive learning across different augmentations of the same SEM image. These approaches allow the model to learn fundamental features of fibrous structures without any human-provided labels. Once pretrained in this way, the model can be fine-tuned on a small labeled data set for segmentation or measurement, often resulting in improved performance due to the strong initialization.^95,96^ For instance, a self-supervised model might learn the concept of fiber continuity or texture from hundreds of unlabeled images, which then helps it detect faint fibers when fine-tuned for segmentation. In that approach, models are first trained on unlabeled fiber images using proxy tasks (such as predicting missing image parts or distinguishing transformed images) to learn useful representations and then fine-tuned on the actual labeled task. Recent studies in materials imaging have begun to employ self-supervised feature learning methodologies, such as SimCLR and MoCo, on micrograph data sets.^97^ These approaches hold potential for direct application to nanofiber SEM images. This emerging trend is anticipated to alleviate the burden of annotation and facilitate access to deep learning techniques for researchers with extensive image archives but limited labeled data.

#### 3.2.3 Federated Learning for Collaborative Model Training

Nanofiber research is often distributed across many institutions and companies, each holding proprietary SEM image data sets generated from different polymers, processes, or machine settings. Privacy, intellectual property, or confidentiality concerns make it difficult to centralize such data, which, in turn, limits AI model development because individual data sets are typically small. Federated learning (FL) offers a solution: it allows multiple parties to collaboratively train a shared AI model without sharing their raw data. In FL, each site trains the model locally on its own images and sends only model updates (gradients) to a central server (or aggregator), which combines them to improve the shared model. Thus, no confidential images leave the premises.^98^ This approach, widely explored in medical imaging due to concerns about patient data privacy, can also be applied to nanofiber images. For example, one can envision a federation of electrospinning laboratories or manufacturing companies collaboratively training a fiber segmentation or defect-detection model. The resulting model would be much more robust and general, having seen fibers with diverse morphologies, diameters, polymers, and imaging conditions, yet without any lab disclosing its proprietary images. In practice, implementing FL could greatly expand the training data effectively available to AI developers, leading to more general and powerful nanofiber characterization models.

However, practical challenges remain. Some challenges include ensuring consistent labeling across participants and handling differences in image distributions, but ongoing research in FL addresses these (e.g., normalization techniques for data heterogeneity^99^). Nevertheless, as privacy-preserving FL frameworks mature, the next few years may see consortia or multicenter projects leveraging FL to build the next generation of nanofiber AI tools that benefit everyone.

### 3.3 Transformer-Based Models

Transformer-based models, originally developed for natural language processing (NLP), have recently been adapted for vision tasks, giving rise to Vision Transformers (ViTs).^100^ ViTs use self-attention mechanisms to capture long-range dependencies in images, which could be advantageous for analyzing nanofiber networks that have complex, overlapping structures. CNNs rely on local receptive fields and progressively enlarged receptive fields through stacked convolutions, whereas vision transformers model global context and long-range dependencies via self-attention.^101,102^ This ability to integrate global context is particularly promising for nanofiber analysis, where structures are complex, overlapping, and extend across large image regions. For example, ViTs could assess overall fiber alignment, distinguish closely spaced or intersecting fibers, or trace fibers through dense crossings where CNNs might struggle. However, classic ViTs operate on fixed-size patches and lack an innate locality bias, which means that early models required extremely large data sets and careful architectural design for segmentation tasks. To address this, researchers have introduced hybrid models combining CNN backbones with Transformer layers, as well as segmentation-specific ViTs. In materials imaging, pure ViT applications remain limited, but analogous work in medical imaging has shown that Transformers can outperform CNNs in segmenting complex structures by modeling long-range interactions. A hypothetical Vision Transformer for nanofibers could, for example, attend to an entire fiber’s span, aiding in tracing it through dense crossings where a CNN might get confused. As ViTs have begun to match or surpass CNN performance in general vision tasks, the community is looking to apply them for finer tasks like fiber segmentation.^102^

Although domain-specific applications in nanofiber imaging are still scarce, early evidence and insights from related fields suggest that transformer-based approaches—whether pure ViTs or CNN-Transformer hybrids like TransUNet or SegFormer—hold strong potential to enhance fiber segmentation and defect detection as larger annotated nanofiber data sets become available.

Recent developments in vision foundation models have opened new possibilities for microscopy image analysis. The Segment Anything Model (SAM), developed by Meta AI, represents a paradigm shift as a promotable segmentation model trained on over 1 billion masks.^103^ Archit et al. introduced μSAM (Segment Anything for Microscopy), a specialized adaptation of SAM fine-tuned for light and electron microscopy applications.^104^ By training generalist models on diverse microscopy data sets, μSAM achieves high-quality segmentation across different imaging modalities with minimal user interaction (Figure 8). For fiber analysis, foundation models like SAM offer the advantage of zero- or few-shot learning capabilities, potentially reducing the annotation burden that has traditionally limited deep learning adoption in nanofiber research. Hybrid architectures combining CNN encoders with Transformer decoders have shown particular promise for complex fiber networks. TransUNet and Swin-UNet leverage the spatial inductive bias of convolutions for local feature extraction while utilizing self-attention mechanisms for global context modeling.^105^ Hybrid CNN–Transformer segmentation models are promising for complex microscopy problems because they combine local convolutional feature extraction with global context modeling.^105,106^

**Figure 8:**
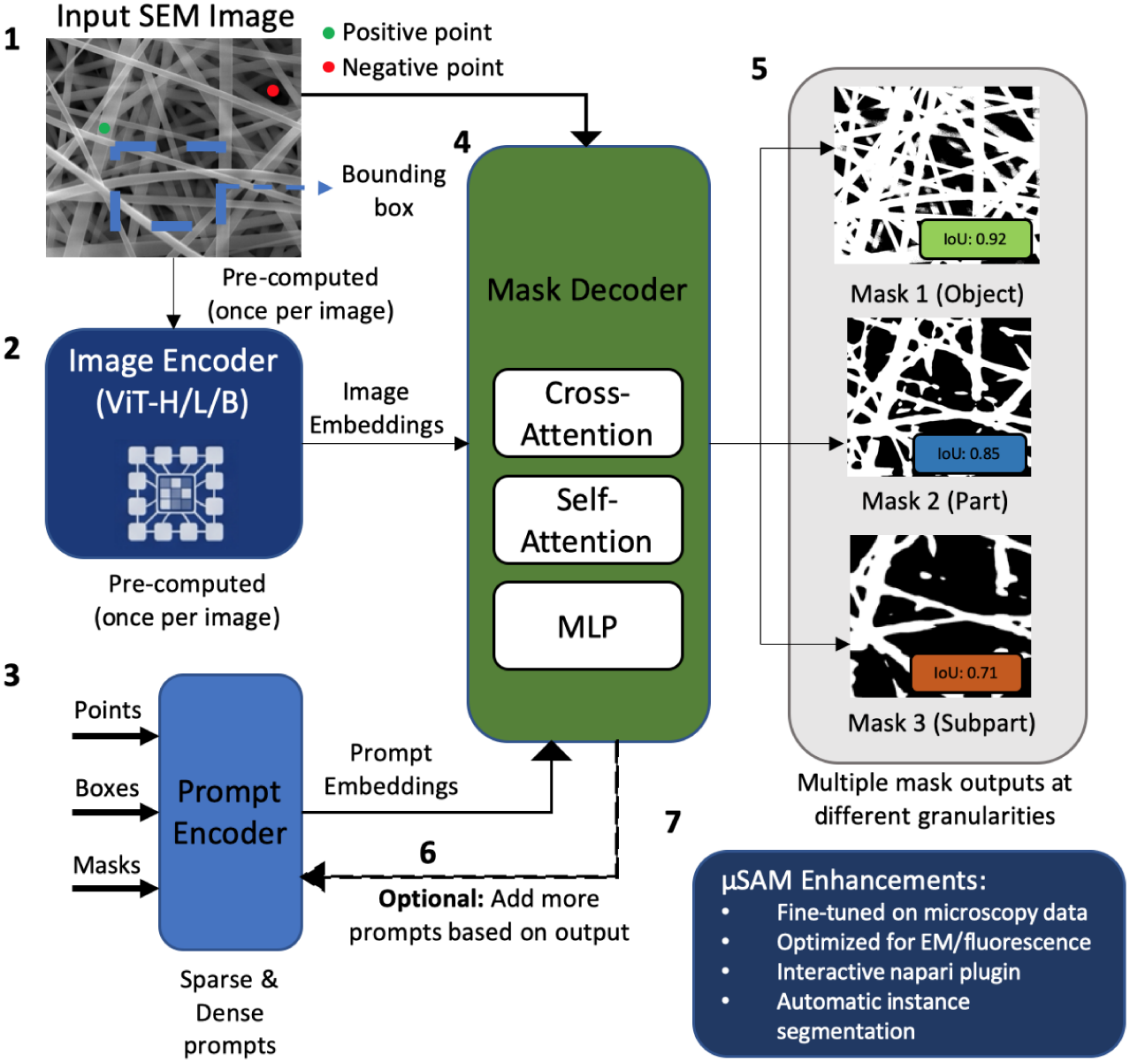
Segment Anything Model (SAM) workflow for interactive nanofiber segmentation. SAM accepts flexible prompts including points (positive for target, negative for background), bounding boxes, or masks from previous iterations. The image encoder (based on Vision Transformer) produces image embeddings, while the prompt encoder processes user inputs. The lightweight mask decoder combines these to generate segmentation masks with associated confidence scores.

### 3.4 Generative AI

Generative AI techniques, particularly generative adversarial networks (GANs) and diffusion models, are increasingly being explored for nanofiber analysis due to their dual role in image enhancement and synthetic data generation. GANs pit a generator against a discriminator in a competition, enabling the generator to produce images that mimic the real data distribution. In materials science, GANs have been used to synthesize realistic microstructure images for augmentation.^107^ For electrospun fibers, GANs can create SEM-like images with controlled diameters, orientations, and alignment patterns, thereby expanding training data sets and alleviating the data scarcity that often limits deep learning applications.^7^ Chun et al. demonstrated a GAN capable of generating porous, energetic material microstructures statistically indistinguishable from real samples.^107^ GANs have also been adapted for image enhancement tasks, such as super-resolution to improve SEM clarity or denoising to reduce charging artifacts. While specific studies on GAN enhancement of nanofiber images are still emerging, the success of GANs in analogous domains (such as enhancing low-light microscopy images) suggests they can improve nanofiber image clarity and thereby measurement accuracy. Nonetheless, GAN training can be unstable and may struggle to capture very fine fiber details without artifacts.

Diffusion models represent a newer class of generative architecture that iteratively refine random noise into high-fidelity images and have recently surpassed GANs in image synthesis quality.^108^ Although there is little published on diffusion models specifically for nanofiber images yet, their potential is significant. They could be used to generate synthetic nanofiber structures with even greater realism than GANs or to perform image enhancement tasks such as super-resolution, denoising, and reconstruction. For instance, diffusion models for super-resolution have achieved impressive results on natural images, and a similar strategy could enhance SEM images of fibers by generating plausible high-frequency details. Likewise, inpainting with diffusion models could reconstruct missing regions of a fiber mat image—useful when parts of the image are occluded or damaged—while preserving realistic fiber textures. Beyond these tasks, diffusion has also been applied to general image restoration;^109^ applying this to nanofibers could help correct imaging artifacts or adjust contrast in a learned manner.

Overall, while transformer and diffusion models are cutting-edge and not yet commonplace in nanofiber metrology, they represent the next wave of AI techniques. By generating realistic synthetic fibers and enhancing image quality, these models can both expand training data sets and improve measurement accuracy. Looking forward, hybrid approaches (e.g., combining a CNN or U-Net with a vision transformer module for improved segmentation or using GANs/diffusion models to generate training data and enhance images) will play an increasingly important role as these technologies mature.

### 3.5 AI-Assisted Image Processing for Nanofiber Analysis

Before the advent of deep learning, automated image processing techniques were developed to extract nanofiber metrics from microscopy images. Many of these techniques remain useful and are often integrated with AI methods. Key steps include edge detection, contour extraction, morphological operations, and image enhancement, which can be applied individually or as preprocessing for ML models.

#### 3.5.1 AI-Driven Noise Reduction and Contrast Enhancement

High-quality image preprocessing can significantly improve analysis outcomes. Noise in SEM/TEM images (e.g., from sensor or environmental sources) can cause false edges. Traditional filters (Gaussian blur, median filter) are commonly applied to smooth the image before segmentation. Now, AI techniques offer more advanced denoising—for instance, convolutional autoencoders can learn to map noisy inputs to clean outputs by training on image pairs. Similarly, contrast enhancement can be achieved with adaptive histogram equalization or with more novel approaches, such as GAN-based enhancement. A GAN trained to translate low-contrast images to high-contrast versions can vastly improve fiber visibility. For example, generative models have been developed to enhance low-light or low-contrast images by learning the specific noise patterns and illumination issues.^110^ Similarly, the diffusion-based enhancement approach can be used for denoising. By essentially learning the distribution of clean nanofiber images, a diffusion model can iteratively improve a degraded image.

#### 3.5.2 AI-Based Measurements

The deep learning approach by Huarachi et al. essentially built the distance transform into the network—their U-Net outputs a distance map image where the intensity corresponds to the fiber radius.^7^ This simplifies diameter computation since each pixel’s value (in the output) directly gives the distance to the nearest edge. Such end-to-end AI approaches avoid the need for separate skeletonization and distance-transform steps, potentially reducing errors at junctions.

For porosity estimation, image analysis involves identifying pore regions (either explicitly segmenting pores or implicitly via a fiber mask). A simple metric is area porosity: the total area of voids divided by the total area. More complex is pore size distribution, which can be obtained by measuring the areas of connected void regions in the binary complement of the fiber mask.^9^ Traditional methods might apply algorithms such as the maximum inscribed circle to each pore to get an effective pore diameter. AI can improve this by accurately segmenting pores even when they have irregular shapes or when the contrast is low. Some studies use pixel clustering or segmentation to identify interfiber pores; for example, an unsupervised learning method might cluster pixel intensities to separate solid vs void. With deep learning, one could train a model to label each pixel as fiber, pore, or background if needed (making it a multiclass segmentation). The output could directly highlight the pore regions for measurement.

AI has also been used for fiber orientation and alignment detection: traditionally, the Fast Fourier Transform (FFT) of the image is used to identify the dominant fiber orientation (peaks in the frequency spectrum correspond to alignment). Now, CNNs can learn orientation directly by regression or classification. Yaman et al. (2021) used automated image analysis to quantify the alignment fidelity of gold nanorods on protein nanofibers, combining ML and image processing to assess how well nanorods followed the fiber direction.^111^ Techniques like the structure tensor or orientation histogram (from skeletonized fibers) are still used, but AI could potentially classify an entire image as “aligned” or “random” and even output an orientation distribution.

#### 3.5.3 Explainable AI (XAI) for Transparency

As AI models become integral to nanofiber characterization, quality control, and materials evaluation—particularly in high-stakes domains such as biomedical scaffolds and high-value manufacturing—trust and transparency are essential. Explainable AI (XAI) techniques aim to make these black box models more interpretable, providing insight into why a model gave a particular output. In nanofiber characterization, XAI could be applied to, for example, highlight which regions of an image led an AI model to flag a defect or how the model decided a fiber is “beaded” vs “smooth”. Methods like saliency maps, Grad-CAM, or feature attribution can overlay a heat map on the fiber image to show areas that most influenced the AI’s decision.^82^ This transparency is critical: a technician might not accept an AI verdict of sample fails quality check without explanation, but if the system indicates these five locations have abnormally large pore sizes (bright red on the heatmap), which drove the fail decision, the justification can be verified and trusted.

XAI also assists developers in debugging models, ensuring they focus on fiber-relevant features rather than spurious background patterns. If AI measurements diverge from classical ones, explanation tools such as saliency maps or Grad-CAM can help reveal which image regions and boundaries drove the model’s prediction.^79–82^ XAI visualizations could reveal that the model indeed perceives this halo as part of the fiber. Similarly, in segmentation tasks, XAI might visualize feature maps to show which structures the network recognizes as fiber, helping to ensure that trivial artifacts are not misclassified as defects.

Recent materials science literature emphasizes integrating domain knowledge into AI and providing explanations.^80^ For example, an XAI approach could be used to ensure a porosity prediction model bases its predictions on visible pore structures rather than spurious correlations like image brightness. Some researchers have started creating explainable models for microstructure–property relationships. Pilania et al provided a framework for explainable machine learning in materials informatics.^81^ By applying these ideas, nanofiber AI systems can be developed that quantify the contribution of fiber diameter uniformity to alignment and produce human-interpretable properties (e.g., porosity is high, alignment is low, therefore quality = X). Additionally, simpler surrogate models or rule extraction from neural networks might be used to validate that AI measurements conform to known physics (for instance, verifying that measured fiber diameter distributions from the model follow expected trends when process parameters change).

The importance of XAI is emphasized across many application domains because users are more likely to trust and adopt models whose reasoning can be examined and related to domain knowledge.^79,80,82^ In manufacturing, this is particularly true—plant managers and engineers are more likely to deploy AI if it comes with explanations. Overall, incorporating XAI will be key to integrating AI seamlessly into nanofiber characterization workflows. By providing human-understandable outputs alongside predictions, future nanofiber AI models can move from being mysterious oracles to transparent assistants, making end-users and industry stakeholders far more likely to trust and deploy them.

#### 3.5.4 Multiparameter AI Characterization

Modern AI-based image analysis enables the simultaneous extraction of multiple morphological parameters from nanofiber SEM images, moving beyond single-metric characterization to provide a comprehensive structural assessment.

AI-based porosity estimation typically employs semantic segmentation to classify each pixel as either fiber or pore and then calculates the area ratio. Image-based porosity estimation typically starts with segmentation of fiber and void regions, followed by area-ratio calculation. In electrospun veils, image-derived digital porosity has been shown to be a feasible and rapid image-analysis-based metric.^9^ Cuahuizo-Huitzil et al. (2024) demonstrated that image-derived digital porosity correlates strongly (*R* ^2^ > 0.90) with conventional gravimetric measurements, validating AI-based porosity estimation as a rapid, nondestructive alternative.^9^ Advanced approaches incorporate 3D reconstruction from multiple 2D images or tomographic data to estimate volumetric porosity, accounting for through-thickness variations not captured in single-image analysis.

Instance segmentation methods can identify and measure individual pores within nanofiber mats. After segmenting the void space, connected component analysis or watershed algorithms separate overlapping pores, and morphological descriptors (area, equivalent diameter, aspect ratio) are computed for each pore. When overlapping structures must be separated before downstream analysis, instance-aware and promptable segmentation models are valuable; FibeR-CNN improved fiber-object segmentation over standard Mask R-CNN on fiber images,^112^ and μSAM improved segmentation quality across diverse light- and electron-microscopy conditions after fine-tuning.^104^

Fiber orientation analysis traditionally relies on fast Fourier transform (FFT) or structure tensor methods applied to skeletonized images. AI-enhanced approaches use CNNs to directly regress orientation distributions or classify images by alignment quality. OrientationJ, bundled with DiameterJ, provides automated orientation histogram generation,^8^ while more advanced methods train neural networks to predict orientation parameters without explicit skeletonization.^94^

Structural anomalies, including beads, fiber breakage, and nonuniform regions, can be automatically identified using classification or anomaly detection networks.^77^ More recent approaches employ unsupervised anomaly detection, training generative models on defect-free images and flagging deviations from the learned distribution. In Table 2, AI models for nanofiber characterization are compared, and Figure 9 shows the decision tree used to select the methods elaborated.

**Table 2:** Comparative Summary of AI/ML Methods for Nanofiber Characterization

AI Model	Category	Primary Task	Performance & Characteristics	Ref.
Random Forest (RF)	Classical ML	Diameter prediction/quality prediction	Direct electrospun evidence: ensemble-tree regression/classification. On a literature-derived electrospinning data set, RF achieved test-set *R* ^2^ = 0.9468 and RMSE = 92.3 nm and outperformed the other evaluated ML models; the underlying algorithm is defined in.	^85,113^
Multilayer Perceptron (ANN)	Neural Networks	Process Modeling	Direct electrospun evidence: predicted PAN nanofiber diameter from spinning-solution concentration, spinning voltage, collecting distance, and solution flow rate; reported comprehensive fit coefficient 0.98952 and average error of 2%.	^33^
Autoencoder + MLP (AE-MLP)	Hybrid ML	Defect/anomaly classification	Direct electrospun evidence: hybrid unsupervised-supervised pipeline for defective vs nondefective SEM nanopatches; reported accuracy up to 92.5% with reduced model complexity relative to heavier CNN-based strategies.	^77^
U-Net	Semantic Segmentation	Fiber/background separation and diameter-map regression	Direct fiber/electrospun evidence: encoder-decoder architecture with skip connections. Modified U-Net/U-Net-regression workflows reported low error in automatic fiber-diameter computation from micrographs (e.g., MAE 0.1094 and MSE 0.0711 on the real test subset) and support multi-image batch sizing.	^7,35,78^
Transfer-learning CNNs (ResNet/GoogleNet)	CNN	Morphology classification	Direct electrospun evidence: transfer learning on electrospun-fiber morphology images markedly improved classification; fine-tuned ResNet reached 95.21% test accuracy and GoogleNet 96.34%, versus 79.35% for ResNet without transfer learning.	^94,114^
FibeR-CNN	Instance Segmentation	Individual fiber isolation and size distribution	Direct fiber-image evidence: instance segmentation model extending Mask R-CNN with additional heads for width and length prediction; surpassed standard Mask R-CNN by 33% in mean average precision (11 percentage points) and predicts fiber width/length distributions.	^112^
SAM/μSAM	Foundation Model	Promptable/few-shot segmentation	Transferable microscopy evidence: SAM is a promptable segmentation foundation model; μSAM adapts it to light and electron microscopy and reports improved segmentation quality across diverse imaging conditions with both interactive and automatic use.	^103,104^
Conditional GAN (c-GAN) + Transfer Learning	Generative AI	Synthetic SEM generation and anomaly classification	Direct electrospun evidence: c-GAN generated synthetic defective/nondefective electrospun-nanofiber SEM images and a transfer-learning classifier validated on real images reported accuracy up to 95.31%.	^115^
Vision Transformers (ViTs)	Transformer	Global-context feature modeling and segmentation backbones	Method-level/emerging evidence: self-attention-based architectures capture global context and long-range dependencies; in this review they are best framed as emerging backbones or hybrid encoders for segmentation rather than fully electrospun-validated standalone methods.	^101,102,105^
Denoising Diffusion Probabilistic Models (DDPMs)	Generative Models	Synthetic data generation/restoration/enhancement	Method-level/emerging evidence: generative diffusion models learn data distributions through iterative denoising; in the present nanofiber context they are best treated as promising tools for synthetic image generation and image restoration/enhancement, not yet as established electrospun benchmarks.	^109,116^

**Figure 9:**
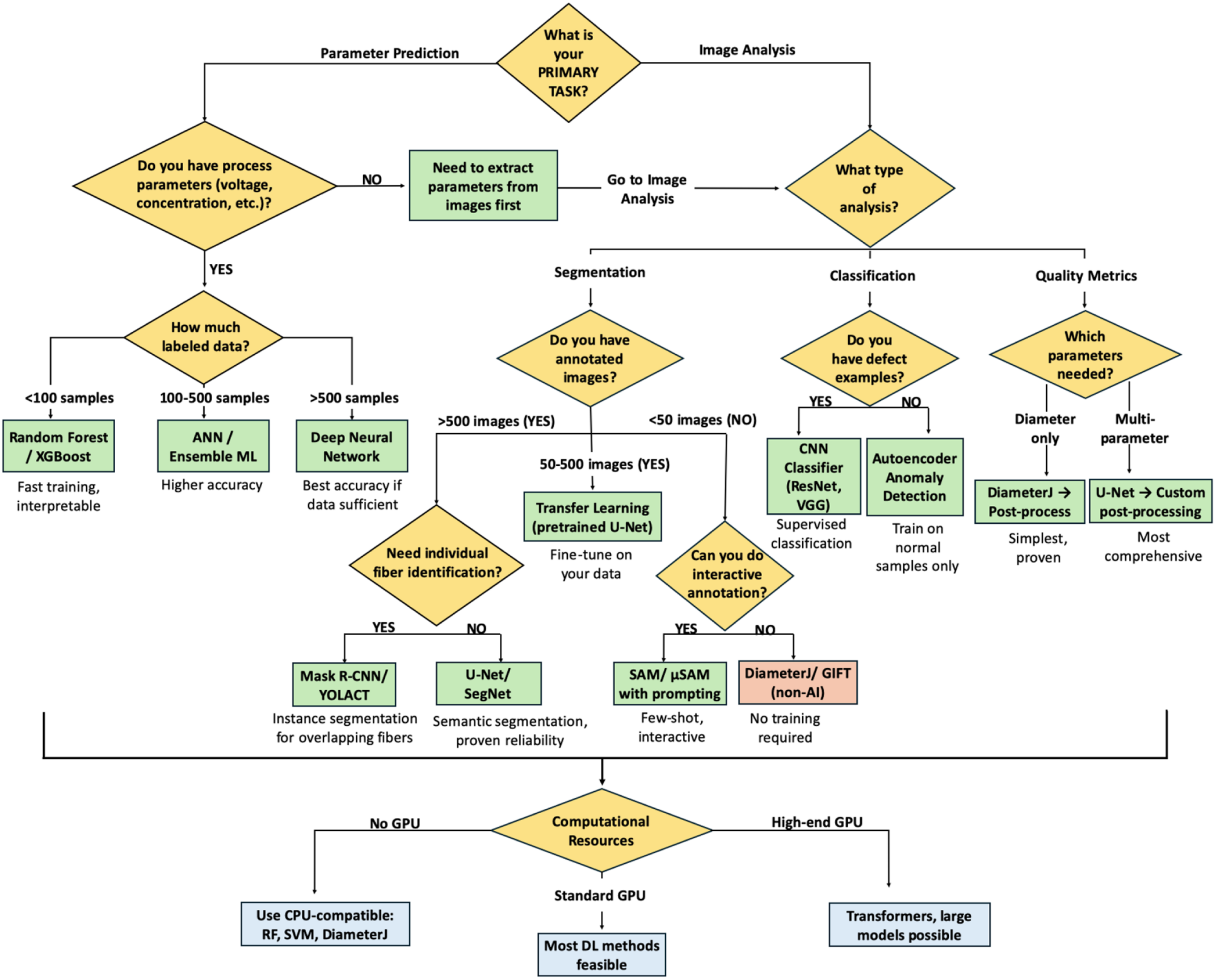
Decision tree for selecting appropriate AI methods for nanofiber characterization. Starting from the primary task requirement, the tree guides users through key decision points, including available data, computational resources, and specific application needs, to recommend the most suitable approach. Leaf nodes indicate recommended methods with brief justifications.

## 4 Benchmarking and Public Data Sets

To evaluate and compare AI models for nanofiber characterization, researchers rely on various data sets and performance metrics. However, the field lacks a large, standardized public database dedicated to nanofiber images, mainly because of the niche application and the effort required to annotate fiber structures. Despite this, a few data sets have been made available and serve as useful benchmarks.

### 4.1 Nanofiber SEM Image Sets

Carrera et al. (2016) published a data set of 45 SEM images of electrospun polycarbonate nanofiber mats, with expert annotations of defective regions. This data set, associated with their defect detection study, has been used in subsequent work on anomaly detection. It provides labels for “normal” vs “defect” pixels, allowing supervised training and testing of classification/segmentation models. The images vary in magnification and fiber density, making them a challenging benchmark for general defect-detection algorithms. The authors made the data set public to encourage comparisons—indeed, their paper reported an algorithm and baseline accuracy that later methods have aimed to beat.^117^

### 4.2 DiameterJ Validation Data Set

During the development of DiameterJ, Hotaling et al. compiled a set of synthetic and real images for validating fiber-diameter measurements.^8^ They generated digital images of fibers with known diameters (white lines on a black background) and included SEM images of fibers with known reference diameters (e.g., calibrated fibers or wires). Although not widely used as a standalone benchmark, this data set (published in a Data in Brief article) is useful for testing the accuracy of diameter measurement algorithms.^118^ Any new diameter measurement method can be applied to these images to directly compare measured vs true diameters. The DiameterJ data set is relatively small, but it is one of the few data sets with ground-truth diameters.

### 4.3 Synthetic Fiber Image Libraries

To mitigate data scarcity, researchers have created synthetic data sets using simulation. For example, Huarachi et al. generated a large set of simulated fiber micrographs with random numbers of fibers, orientations, and noise levels. These synthetic images (with known ground truth masks) effectively serve as an unlimited data set for training and evaluating segmentation models. Some of these libraries have been open-sourced or described in publications so that others can regenerate them. While not real data, they are incredibly valuable for benchmarking due to the perfectly known ground truth. A model’s performance can be quantified in terms of pixel-wise accuracy or Dice coefficient against the known mask. In Huarachi’s study, they organized images into groups for training and testing (e.g., G1: real train, G2: real test, G3: synthetic train, G4: synthetic test) to systematically evaluate how models perform on real vs synthetic data. Such benchmarking showed, for instance, that training on a mix of real and synthetic images improved performance on real test images, highlighting the benefit of synthetic augmentation.^7^

While the data sets described above provide useful benchmarks, public challenges or leaderboards specifically for nanofiber analysis have not yet been established, likely due to proprietary data and the specialized nature of the problem. However, as the community grows, we may see the emergence of shared data sets (possibly through initiatives such as the Materials Genome or image databases) and organized challenges. In the meantime, authors are encouraged to release their trained models and data when possible. The few that have (e.g., code on GitHub like the FibeR-CNN expansion of Mask R-CNN^112^) greatly aid reproducibility. Benchmarking a model from one group on another’s data is becoming more common, providing a more objective comparison. For example, a deep model trained on one lab’s electrospun polyvinylidene fluoride (PVDF) fibers might be tested on another lab’s polyacrylonitrile (PAN) fiber images to assess generalization.

### 4.4 Evaluation Metrics

Evaluation metrics used in nanofiber analysis mirror those used in image segmentation and object detection. In benchmarking between architectures, studies often compare a new method against prior methods on a common data set. These benchmarks are critical to justifying the move to AI approaches. Generally, the trend has been that each successive generation of AI models (e.g., going from basic CNN to U-Net, then to advanced architectures) shows improved metrics on the available data sets. Commonly used AI-driven nanofiber analysis evaluation metrics are listed in Table 3.

**Table 3:** Common AI-Driven Nanofiber Analysis Evaluation Metrics

Evaluation metrics	Aimed attributes	Examples
*Pixel-level accuracy* and *Intersection over Union (IoU)*	Segmentation quality	An IoU above 0.90 might be reported for a model that very tightly matches expert-drawn fiber masks.
*Dice coefficient* or *F1-score*	Segmentation quality	Indicating overlap between predicted fiber regions and true fiber regions.
*Mean Absolute Error (MAE)* and *Mean Squared Error (MSE)*	Continuous measurements	If ground truth diameters for each fiber (or distribution statistics like mean diameter) are known, the error of the AI measurements can be computed. Huarachi et al. reported the MAE and MSE of their U-Net’s diameter predictions to demonstrate its accuracy.^7^
*Accuracy, Precision, Recall, and F1*	Classification tasks (defect vs nondefect identification, or classifying images by alignment quality, etc.)	A defect detection model might be evaluated by precision (what fraction of detected defect regions are true defects) and recall (what fraction of actual defects were detected).
*R* ^*2*^ *(coefficient of determination)*	Regression tasks	*R* ^2^ can also be used to assess agreement between image-analysis-based porosity estimates and reference porosity measurements.^9^

## 5 Comparison with Traditional Characterization Methods

It is instructive to benchmark AI-driven methods against classical image processing and manual techniques to quantify the gains in speed, accuracy, reproducibility, and scalability.

### 5.1 Accuracy and Detection Capability

AI models have proven more accurate in identifying fibers and defects than traditional methods. For example, the classical CT-FIRE algorithm (curvelet transform + fiber extraction), which was used for semiautomated fiber segmentation, often fails on complex images—it may miss fibers that overlap or those below a certain length, and it produces insufficient results on challenging data sets.^112^ In contrast, deep learning models like FibeR-CNN detect virtually all visible fibers and can even tease apart overlapping ones by learning their contours.^112^ In defect detection, human inspectors might overlook subtle issues due to fatigue or bias, whereas AI can consistently apply the same criteria across all images.^94^ In fiber diameter measurement, manual or threshold-based methods typically report errors of a few tens of nanometers and can be skewed by subjectivity in threshold selection; the U-Net-based segmentation yields more objective measurements and better agreement with ground truth across a range of images.^78^ Thus, in terms of accuracy, AI-based characterization either matches human-level performance or exceeds it, especially when patterns are complex or faint.

### 5.2 Speed and Throughput

Manual analysis of nanofiber images is extremely slow—a skilled technician might spend 15–30 min per SEM image tracing fibers or measuring diameters using software such as ImageJ. Classical semiautomated tools (e.g., DiameterJ or CT-FIRE) improve speed but still may take on the order of minutes per image for processing, especially if iterative parameter tuning is needed for each image. CT-FIRE, for instance, was reported to be extremely slow (∼[several minutes] per image on a single CPU core).^112^ Once trained, AI methods are remarkably fast at inference. A CNN can process an image in a fraction of a second on a modern GPU or a few seconds on a CPU. This means an entire data set of hundreds of images can be analyzed in minutes, a dramatic improvement. In a production environment, what used to be a bottleneck (stop production, take samples to the lab, analyze for hours) can become near-real-time feedback. The Hong Kong Productivity Council (HKPC) industrial case suggests that continuous monitoring is feasible when inference speed matches production throughput.^119^ Moreover, AI scales linearly; analyzing 100 images is roughly 100× the time of one image, which is trivial when each is a second or less; and adding more computing resources can parallelize this further. On the other hand, adding more images for a human or a semiautomated process might introduce exponential effort (more fatigue, more parameter tweaking). Therefore, in high-throughput scenarios, AI is indispensable.

### 5.3 Reproducibility and Objectivity

Traditional manual measurements are subject to interoperator and intraoperator variability. Two people measuring the same fiber sample might get slightly different diameter distributions due to differences in how they threshold the image or which fibers they choose to measure. Even the same person might get inconsistent results if they are tired. AI methods offer far superior reproducibility: a trained model produces the same output for the same input every time, with no drift. This consistency is crucial for quality control and for research comparisons. Classical image processing pipelines can be reproducible if the parameters are fixed, but in practice, users often adjust settings per image to get a good result, introducing subtle biases. For example, a thresholding method might require adjusting brightness or contrast on an image-by-image basis. AI approaches largely avoid such case-by-case tweaking; a model trained on diverse data knows how to handle typical variations. As noted in one study, classical fiber analysis tools had many parameters that need to be carefully tuned—often on a per-image basis—to optimize results,^112^ whereas a deep network automatically learns robust features, eliminating the need for manual parameter tuning. This objectivity means AI analysis can be validated and then trusted to run autonomously, a key advantage for industry adoption.

### 5.4 Scalability

As nanofiber production ramps up and experimental studies involve larger data sets (e.g., capturing an entire roll of nanofiber mat or doing high-throughput experiments with many samples), the methods must scale. Manual methods clearly do not scale—the labor cost and time become prohibitive. Traditional software might scale to a point, but memory and computational constraints, or the aforementioned need for parameter adjustment, often limit its utility on very large data sets. AI solutions, by virtue of their algorithmic efficiency in inference, can handle large volumes of data. In addition, AI models can be trained to handle variations (e.g., different materials, imaging conditions), making a single model applicable to many scenarios, whereas classical methods might require reconfiguration for each new scenario. There are also frameworks for distributed or cloud-based AI processing, meaning multiple machines can share the load of analyzing terabytes of image data—something not straightforward with interactive manual analysis. In summary, the scalability of AI enables handling the big data aspect of modern nanomaterials research and manufacturing, turning what was once a painstaking sampling process into a comprehensive analysis of entire data sets.

### 5.5 Addressing Traditional Limitations

Many limitations of traditional characterization are effectively tackled by AI. For instance, poor image quality (noise, low contrast) would stump simple edge detectors or threshold methods, but a CNN can learn to see through noise by training on examples with variation—effectively acting as a denoiser and segmenter in one. Irregular or complex fiber layouts (nonuniform backgrounds, fibers crossing) would break the assumptions of algorithms that expect isolated objects, but AI handles complexity by learning from it. And tasks that were simply not feasible before, like predicting mechanical properties directly from images, become attainable with AI models that can find hidden correlations. Controversially, AI is not completely infallible; if it encounters data that are completely outside its training distribution, it may misclassify or segment incorrectly. With careful training and validation, its performance within the expected domain far exceeds the old methods. Researchers have found that even when they deliberately introduced variations, AI models were robust: e.g., a model trained on synthetic images still worked on real images with different contrast,^120^ showing flexibility that no hand-crafted method could match.

In quantitative terms, the literature consistently reports higher accuracy metrics (IoU, F1 scores for segmentation, classification accuracy, etc.) for AI methods over traditional ones.^94^ Combined with the qualitative benefits of speed and consistency, AI-based nanofiber characterization clearly outperforms legacy methods. The main cost—preparing a training data set and training the model—is increasingly mitigated by transfer learning, synthetic data, and collaborative efforts, as discussed above. Any remaining limitations of AI (like interpretability or the need for large training data) are being addressed by emerging techniques such as explainable AI and federated learning. Table 4 exemplifies the role comparison of manual, conventional, ML, and DL methods in nanofiber diameter analysis.

**Table 4:** Comparison of AI-Based (Machine Learning and Deep Learning) and Traditional Methods (Manual Measurement and Conventional Methods) for Nanofiber Diameter Analysis

Metric	Manual Measurement	Conventional/Semi-Auto (e.g., SIMPoly/DiameterJ)	Machine Learning (e.g., RF/ANN)	Deep Learning (e.g., U-Net/FibeR-CNN)
**Measurements per Image**	25–100 (Limited)	>1000 (Statistical)	Parameter-based prediction	Full Pixel-level Analysis (∼3M+ pixels)
**Analysis Time**	15–60 min	20–120 s	<1 min (Once trained)	0.5–10 s (GPU accelerated)
**Measurement Accuracy**	Baseline (Subjective)	High (If binarization is perfect)	Very high	State-of-the-Art (High IoU/mAP)
**Handling Overlaps**	Poor/Subjective	Prone to errors at intersections	Indirect/Pattern-based	Excellent (Specifically optimized)
**Operator Dependency**	High (High bias)	Medium (Sensitive to thresholds)	Low (Consistent results)	None to Low (Automated pipeline)
**Defect Detection**	Manual Visual Inspection	Limited (Pattern-based)	Good (Feature classification)	Excellent (Automated identification)
**Training Needs**	None	None	100–500 Data Samples	100–1000+ Images (SAM: < 50)
**Primary Limitation**	Low throughput & Fatigue	Sensitive to noise/resolution	Requires structured input data	High computational (GPU) cost
**References**	^39^	^29,76^	^33,85^	^112^

## 6 Industry Applications and Smart Manufacturing

Beyond academic research, AI-driven nanofiber characterization is increasingly finding its place in industrial applications, particularly under the umbrella of smart manufacturing and Industry 4.0. Manufacturers of nanofiber-based products (filters, textiles, medical mats, etc.) are beginning to integrate AI for real-time monitoring and quality control on production lines.

### 6.1 Real-Time Quality Control in Electrospinning

One of the most valuable uses of AI in industry is to perform live inspection of fibers as they are produced (for example, in an electrospinning machine). High-speed cameras or imaging sensors can be placed to observe the fiber deposition on the collector, and AI vision models analyze these images on-the-fly. Deep learning algorithms can instantly detect defects such as the formation of beads, nonuniform fiber diameter, or uneven fiber deposition. This real-time analysis allows the production system to adjust parameters in a feedback loop—for instance, if the AI detects a sudden increase in fiber diameter (perhaps due to a drop in voltage or change in solution viscosity), the system could automatically tweak the spinning voltage or feed rate to compensate.^121^ Such AI-driven control helps maintain consistent fiber quality. In addition, real-time anomaly detection means that if a serious issue occurs (e.g., a spinneret clog leading to no fiber output or a drastic misalignment), the system can immediately alert operators or even pause the process to prevent wasted material. The net effect is a reduction in defects and more uniform product quality, achieved with minimal human intervention.

Although reported for optical micro/nanofiber fabrication rather than electrospinning, this study illustrates how inline diameter measurement and feedback control can be implemented in fiber-manufacturing systems.^122^ While this was for optical microfibers, the same concept applies to electrospinning: a camera or inline microscope could capture fiber deposition in real time, an AI model could analyze fiber thickness and alignment, and adjustments to parameters (voltage, collector speed) could be made immediately to correct any deviations. The high speed of inference of modern models (some can exceed 100 frames per second on modest hardware) makes this plausible. This closes the loop for a smart manufacturing setup, reducing waste and ensuring consistent product quality. It is anticipated that further research on lightweight models or field programmable gate array (FPGA)/embedded deployment will facilitate on-machine analysis. Additionally, the combination of AI with robotics (for automated sample handling and imaging) could enable fully automated quality control stations: samples of nanofiber mats could be imaged, analyzed by AI (for defects, uniformity, etc.), and decisions could be made without human involvement.

A notable industry example is the collaboration between the HKPC and a nanofiber manufacturer (Nanoshields Technology Ltd.) to create smart electrospinning production lines.^119^ In 2022, HKPC custom-designed two automated nanofiber production lines equipped with advanced AI, IoT sensors, and robotics for Nanoshields. Each production line has integrated machine vision and sensor systems that collect environmental and process data (like humidity, voltage, and solution flow rate) in real time. AI models analyze the streaming data to predict product quality metrics and detect anomalies. For example, computer vision monitors the fiber mat formation; if the fiber layer density deviates from the target (indicating a potential thinner or thicker area), the system flags it. The real-time monitoring system visualizes production data and uses AI predictions to shorten response time to quality issues. According to HKPC’s public case report, the implementation was associated with reported reductions in manpower and quality-control burden and with higher output.^119^ This case demonstrates that AI is not just in an isolated lab setting but is deeply integrated into manufacturing—it is analyzing fibers on the fly and making decisions that maintain high-quality standards. Essentially, the electrospinning machine has become smart, adjusting to ensure fiber uniformity and alignment as it produces material, with AI acting as the brain behind the control system.

### 6.2 Automated Optical Inspection Systems

In industrial nanofiber production (e.g., for filters) after the fibers are produced and collected as a mat or membrane, automated inspection is crucial. AI-based inspection systems use cameras or microscopes to scan the finished rolls or sheets of nanofiber material. Computer vision models then evaluate key quality metrics such as fiber uniformity, pore size distribution, weight distribution, and the presence of defects (contamination, broken fibers, etc.). Unlike human inspectors, AI systems can examine 100% of the product at high speed, ensuring no defect goes unnoticed. One practical example is the use of desktop SEMs with automated analysis software in quality laboratories. Commercial systems such as Phenom’s FiberMetric are marketed for automated measurement of fiber diameter, orientation, and pore-related descriptors from SEM images.^123^ This system is touted as operator-independent and fast, providing statistically significant data within minutes. While FiberMetric’s underlying algorithms are proprietary (possibly based on traditional image processing), it reflects industry’s recognition of automated characterization. Newer AI-powered inspection systems take this further by leveraging deep learning to handle greater image variability. For example, AI can robustly measure fibers even when image contrast varies or minor impurities are present, whereas fixed algorithms might fail. In the production of fiber filters, companies have started using vision systems on the line to check fiber layer uniformity. If the AI finds a section with too sparse fibers (which could compromise filtration efficiency), that segment can be marked or culled. In textile nanofibers, AI inspection can ensure that fiber webs meet alignment specifications (important for anisotropic mechanical properties) by analyzing fiber orientation distributions across the roll.

### 6.3 Process Monitoring and Mechanical Property Prediction

AI is also being explored to correlate the observed fiber structures with end-use performance, essentially creating predictive models for material properties. In a manufacturing context, once an AI model has segmented fibers and extracted metrics (fiber diameter distribution, pore size, orientation distribution, etc.), those metrics can be fed into predictive algorithms (which could be machine learning regression models) to estimate properties like tensile strength, filtration efficiency, or breathability of the mat. For instance, if a certain level of fiber alignment is known to enhance tensile strength in one direction, the AI system can quantify alignment in real time and predict if a given batch will meet the mechanical specifications. Researchers are actively working on such structure–property prediction models. A recent study in composite materials showed that a CNN could predict mechanical stress–strain curves from microstructure images.^124^ In nanofibers, similar approaches are under development, where AI bridges the gap from imaging to performance, enabling truly smart manufacturing—not only detecting defects but also ensuring the final product functions as intended.

Overall, industry adoption of AI for nanofiber characterization is on the rise. From intelligent electrospinning lines that self-correct to automated vision inspection replacing tedious manual checks, AI contributes to higher efficiency, lower costs, and improved product consistency. These smart manufacturing implementations build on algorithms validated in research and apply them at scale.

## 7 Conclusion and Recommendations

In conclusion, AI-driven approaches have revolutionized nanofiber characterization over the last five years, delivering significant gains in efficiency, accuracy, and capability. From advanced CNN and transformer models that precisely measure and detect features, to generative models that synthesize training data, and to deployment in smart factories for real-time quality control, the progress has been remarkable. AI methods address longstanding limitations of manual and classical techniques, enabling high-throughput, consistent analysis that keeps pace with modern nanofiber production. Looking forward, emerging AI innovations—transformers, diffusion generators, federated collaborative training, and explainable AI—promise to further enhance how we analyze nanostructured fibers, making the tools more powerful, generalizable, and trustworthy. The synergy of materials science and artificial intelligence is thus driving a new era of intelligent nanofiber manufacturing and research, where data inform decisions at every step and the nanoscale world can be quantified with unprecedented clarity. AI techniques for nanofiber characterization continue to evolve rapidly. Several promising advances and emerging trends are poised to further enhance capabilities in the near future.

### 7.1 Summary of Findings

For routine single-parameter characterization of clear fiber-backgrounded SEM images, conventional automated tools remain the first recommendation. These tools have an ease of accessibility, zero training overhead, and validated performance. Classical machine learning models (random forest and SVM) can be used when conventional tools yield demonstrably unreliable segmentation (fragmented or merged fibers). Deep-learning methods should be reserved for the most challenging scenarios, for instance, high-throughput industrial inspection or situations where the data make manual or semiautomated methods infeasible. In all cases, cross-validation of AI-derived measurements against a subset of conventional or manual measurements is advisable to ensure consistency and trust the results.

AI-driven image analysis brings significant benefits to nanofiber research. Deep learning segmentation (e.g., U-Net, Mask R-CNN) provides pixel-precise fiber extraction even in challenging images with dense or overlapping fibers.^6,7^ This allows for more accurate diameter and pore measurements than semiautomated thresholding techniques. Machine learning models can classify and detect defects with high confidence, reducing reliance on subjective human inspection.^77^ Generative models (GANs, diffusion models) enhance data availability by producing realistic synthetic fiber images for training^100^ noising and super-resolution. Transformer-based architectures, though in early stages for this field, are poised to further improve segmentation by capturing the global context of fiber networks.^102^ Benchmark comparisons show AI methods achieving excellent agreement with ground truth (often within a few percent errors for diameters^8^ or >90% IoU for segmentation) where manual methods struggle. Moreover, AI provides consistency and speed unattainable by human means—measurements that took hours can be done in seconds with minimal variability.^8^ These advantages are driving the adoption of AI in both academic research and industrial settings for nanofiber manufacturing.

### 7.2 Best Practices

Based on the literature, it is recommended to consider the following for AI applications in nanofiber characterization: (1) *Leverage hybrid approaches:* combine AI segmentation with physics-based calculations for metrics (for example, use a CNN to get the fiber mask and then use conventional geometry to compute diameters and porosity). This takes advantage of AI’s strengths while keeping results interpretable in physical terms. (2) *Augment training data:* utilize synthetic fiber images and data augmentation (rotations, noise, intensity shifts) to improve model robustness.^7^ Nanofiber morphology can vary widely; a model exposed to many variations generalizes better to new samples. (3) *Cross-validate with traditional measurements:* especially in critical applications, validate AI measurements against a subset of manual measurements or known standards to build confidence (for instance, check that the average fiber diameter from the model matches what one would estimate with ImageJ manually on a few images). (4) *Tune models to the imaging modality:* SEM, TEM, and optical images of fibers have different characteristics (contrast, resolution). Network architecture and preprocessing should be optimized accordingly—e.g., TEM images might need models that handle low contrast, while SEM images might benefit from networks that can learn complex backgrounds. (5) *Consider transfer learning:* if you have limited labeled data for your specific fibers, consider fine-tuning a model pretrained on other fibrous images or related textures. This can significantly boost performance with few training samples.

### 7.3 Future Outlook and Research Directions

Beyond traditional metrics, AI may unlock new insights in nanofiber research, for example, predictive modeling—using AI to predict mechanical or functional properties of a fiber mat directly from image features. If enough data linking image-derived features to properties (e.g., tensile strength, filtration efficiency) are available, one could train surrogate models to predict performance from an SEM image. This would greatly accelerate R&D feedback loops. Another emerging application is anomaly detection in a one-class learning sense: using generative models or autoencoders to learn what normal nanofiber mats look like and flag any deviations without explicit defect labels (useful for catching unknown defect types or process drifts). This has been touched on by a few works (like using autoencoders for unsupervised defect detection^77^) and will be refined further.

In terms of future trends, we expect AI models to become lighter and more accessible. Techniques such as knowledge distillation and model quantization can reduce the model size, which is helpful for deployment on portable devices or lab-on-chip systems. There is also a trend toward open-source tools and platforms for materials image analysis (for example, libraries that include pretrained models for common tasks). As these tools incorporate the latest AI models, nanofiber researchers will be able to apply sophisticated analyses without deep AI expertise. This democratization will spur wider adoption.

Finally, collaboration between domain experts and data scientists will grow. The nuances of nanofiber production (e.g., how humidity affects fiber morphology) can be incorporated into AI models, either through custom loss functions or by constraining models with physical knowledge (physics-informed AI). For example, knowing that fibers should be continuous, one could enforce continuity in a segmentation prediction. Such physics-informed or knowledge-guided AI will ensure models make physically reasonable interpretations of images, which is a fertile area of research.

Several challenges and opportunities were identified for further studies. One challenge is improving model generalization: a network trained on one fiber type or imaging condition may falter on another. Future research should focus on domain adaptation techniques to make models more universal or on building comprehensive training sets that span the diversity of nanofiber materials and imaging setups. Another area is the development of standards and benchmarks: the community would benefit from an open benchmark data set (or a few data sets representing different fiber scenarios) and standard evaluation metrics to enable fair comparisons of algorithms. This could go hand in hand with an open competition to catalyze innovation. Additionally, integrating domain knowledge (e.g., electrospinning physics) into AI models could constrain solutions to physically plausible ones and reduce data needs. On the interpretability front, more work is needed to create explainable models—perhaps by designing networks that output human-understandable parameters (like fiber density or average orientation) as intermediate steps or by using attention mechanisms that highlight important image regions (letting researchers verify that the model is looking at fibers, not noise).

The current generation of AI-based nanofiber analysis methods has significant limitations that must be explicitly stated. The first is data availability. In practice, comprehensive data sets are often built by combining high-throughput imaging, synthetic data generation, annotation, and augmentation. However, this process requires significant time, expertise, and resources. The second limitation is generalization. Broad data sets help reduce overfitting and improve transferability. But even advanced foundation-model approaches still perform best on data that resemble their training distributions. The third limitation is computational cost. Although inference may be practical in some settings, modern model training and fine-tuning remain hardware-intensive. For these reasons, future research should prioritize openly reusable. Explainable AI should also become a central priority so that future systems not only generate predictions but also indicate when those predictions should not be trusted.

The synergy between AI and nanofiber technologies is still expanding. It is expected that as computing power and algorithms improve, real-time monitoring and control systems will become commonplace, leading to smarter electrospinning machines that can adjust on-the-fly to maintain product specifications. In research laboratories, automated characterization will free scientists to spend more time on designing materials and interpreting results rather than laboring over measurements. Multiscale AI analysis—linking nanoscale fiber features to macroscale membrane performance—is an exciting frontier that could accelerate the development of nanofiber products (from air filters to tissue scaffolds) by rapidly screening candidates via imaging and AI predictions of performance.

In conclusion, AI-based techniques have proven to be transformative for nanofiber characterization, providing rapid, accurate, and rich analyses that extend the capabilities of traditional methods. By embracing these tools and continuing to refine them, the nanofiber community can unlock deeper insights into structure–property relationships and achieve greater control over the fiber fabrication processes. It is recommended that researchers and engineers invest in developing and adopting AI solutions as part of their characterization toolkit, while remaining cognizant of their limitations and thoroughly validating results. With interdisciplinary collaboration—combining expertise in materials science, imaging, and machine learning—the full potential of AI in nanofiber analysis will be realized, driving the field toward more innovative materials and efficient manufacturing processes.
